# Gene expression of muscular and neuronal pathways is cooperatively dysregulated in patients with idiopathic achalasia

**DOI:** 10.1038/srep31549

**Published:** 2016-08-11

**Authors:** Orazio Palmieri, Tommaso Mazza, Antonio Merla, Caterina Fusilli, Antonello Cuttitta, Giuseppina Martino, Tiziana Latiano, Giuseppe Corritore, Fabrizio Bossa, Orazio Palumbo, Lucia Anna Muscarella, Massimo Carella, Paolo Graziano, Angelo Andriulli, Anna Latiano

**Affiliations:** 1 Division of Gastroenterology, IRCCS ‘Casa Sollievo della Sofferenza’, San Giovanni Rotondo, Foggia, Italy; 2 Bioinformatics Unit, IRCCS ‘Casa Sollievo della Sofferenza’, San Giovanni Rotondo, Foggia, Italy; 3 Unit of General Surgery 2nd and Thoracic Surgery, IRCCS ‘Casa Sollievo della Sofferenza’, San Giovanni Rotondo, Foggia, Italy; 4 Medical Genetics Service, IRCCS ‘Casa Sollievo della Sofferenza’, San Giovanni Rotondo, Foggia, Italy; 5 Laboratory of Oncology, IRCCS ‘Casa Sollievo della Sofferenza’, San Giovanni Rotondo, Foggia, Italy; 6 Pathology Unit, IRCCS ‘Casa Sollievo della Sofferenza’, San Giovanni Rotondo, Foggia, Italy

## Abstract

Idiopathic achalasia is characterized by the absence of peristalsis secondary to loss of neurons in the myenteric plexus that hampers proper relaxation of the lower esophageal sphincter. Achalasia can be considered a multifactorial disorder as it occurs in related individuals and is associated with HLA class II genes, thereby suggesting genetic influence. We used microarray technology and advanced *in-silico* functional analyses to perform the first genome-wide expression profiling of mRNA in tissue samples from 12 achalasia and 5 control patients. It revealed 1,728 differentially expressed genes, of these, 837 (48.4%) were up-regulated in cases. In particular, genes participating to the *smooth muscle contraction* biological function were mostly up-regulated. Functional analysis revealed a significant enrichment of neuronal/muscular and neuronal/immunity processes. Upstream regulatory analysis of 180 genes involved in these processes suggested *TLR4* and *IL18* as critical key-players. Two functional gene networks were significantly over-represented: one involved in *organ morphology, skeletal muscle system development and function, and neurological diseases*, and the other participating in *cell morphology, humoral immune response and cellular movement*. These results highlight on pivotal genes that may play critical roles in neuronal/muscular and neuronal/immunity processes, and that may contribute to the onset and development of achalasia.

Idiopathic achalasia is an inflammatory disease of unknown etiology, characterized by the lack of coordinated peristalsis and incomplete relaxation of the lower esophageal sphincter (LES) due to loss of inhibitory nitrinergic neurons in the esophageal myenteric plexus.

Patients present with dysphagia to both solids and liquids, chest pain and regurgitation of undigested food, often leading to weight loss. Due to initial non-specific symptoms in early stage disease and the low prevalence (~1:10000) worldwide, the pathology often is undiagnosed for many years, resulting in the characteristics of the late stage disease. The gold standard for the diagnosis of achalasia is esophageal motility testing with manometry, aided by complimentary tests, such as esophagogastroduodenoscopy and contrast radiography^1^.

Unfortunately, the clinically available pharmacologic therapies for achalasia have limited value. The currently available treatments have the common aim of relieving symptoms by decreasing the pressure of the LES. This can be achieved by botulinum toxin injection, by endoscopic dilation or surgical treatment. Recently, other therapeutic options, including per-oral endoscopic myotomy have been developed and are gaining international consensus^2^.

The likely etiology includes neuronal degeneration, viral infection, genetic inheritance, and autoimmune disease. Current evidence suggests that the initial insult to the esophagus, most likely a viral infection or other environmental factor, causes inflammation in the myenteric plexus and leads to an autoimmune response in genetically susceptible individuals, with subsequent chronic destruction of inhibitory myenteric ganglion cells resulting in the clinical syndrome of idiopathic achalasia^3^.

Although rarely inherited, achalasia is considered a complex disease because familial cases occur^4^,^5^ and the condition is associated with genes involved in autoimmune responses and neuronal function, which suggests genetic influence. Add to this a wide variety of genetic syndromes are associated with achalasia. These include Sjogren’s syndrome, Allgrove’s syndrome (triple “A” syndrome consisting of achalasia, adrenal insufficiency, and alacrima - AAAS), Down’s syndrome, and Parkinson’s disease^1^ A locus on chromosome 12q13 containing a novel gene encoding a 547 amino acid protein named ALADIN was identified in families with Allgrove’s syndrome. AAAS gene mutations generate an abnormal ALADIN protein, which is believed to contribute to the abnormalities of oesophageal myenteric neurons^6^.

Moreover, genetic studies have recently identified HLA alleles^7^,^8^,^9^,^10^,^11^ and single nucleotide polymorphisms in the *PTPN22*^12^, *IL10*^13^, *IL23R*^14^, *VIPR1*^15^, c-*kit*^16^, *IL33*^17^, and *LTA/TNFα*^18^ genes associated with the condition. Recently, a systematic association study^19^, performed on the Immunochip^20^, identified genetic risk factors for achalasia within the HLA-DQ receptor, thereby confirming a key role for immune-mediated processes in the pathogenesis of the disease.

Genome-wide and gene expression studies in achalasia are lacking. There is therefore a pressing need to explore these genetic findings with functional and expression studies so the etiology and pathogenesis of the disease can be better understood and safe and effective treatment developed.

Our study enrolled 12 patients with achalasia and 5 controls. It comes first to analyze the gene expression profiles of achalasia patients. We used bioinformatics and statistical methods to identify critical genes and highly perturbed pathways with potential involvement in achalasia.

## Results

### Clinical data

Table 1 gives the main clinical characteristics of the patients.

The cohort with achalasia consisted of 5 (42%) men and 7 (58%) women, ranging in age from 35 to 77 years (mean 58.5, median 66). The age at disease onset ranged from 17 to 77 years (mean 56.1, median 63.5). The mean LES pressure was 38.8 ± 14.4 mmHg, and dysphagia and regurgitation were present in all patients.

The 5 control patients were all men with a mean age of 66.6 years (median 71) at the time of surgery.

### Microarray gene expression analysis

The ANOVA test between achalasia patients and control samples identified 1,728 differentially expressed genes (DEGs) (min FC −81.1, max FC 35.3, Figure S1). Of these, 837 (48.4%) were up-regulated in patients. The most up-regulated and down-regulated genes are reported in Table 2. Notably, up-regulated genes in patients included the ephrin receptor A7 (*EPHA7*) (FC +35.3, *P* = 1.76 × 10^–8^), and genes involved in smooth muscle contraction: tropomyosin 2 (*TPM2*) (FC +5.33, *P* = 4.05 × 10^–7^) and integrin, alpha 1 (*ITGA1*) (FC +4.12, *P* = 7.09 × 10^–7^). The most down-regulated genes included the chemokine (C-X-C motif) ligand 17 (*CXCL17*) (FC −35.14, *P* = 2.86 × 10^−12^) and the immunoglobulin heavy constant alpha 1 and 2 (*IGHA1-2*) (FC −26.67, *P* = 6.92 × 10^−8^ and FC −32.31, *P* = 1.16 × 10^−8^) genes, which play an important role in innate defense against infections^21^.

### In silico functional enrichment analysis

**Figure 1:**
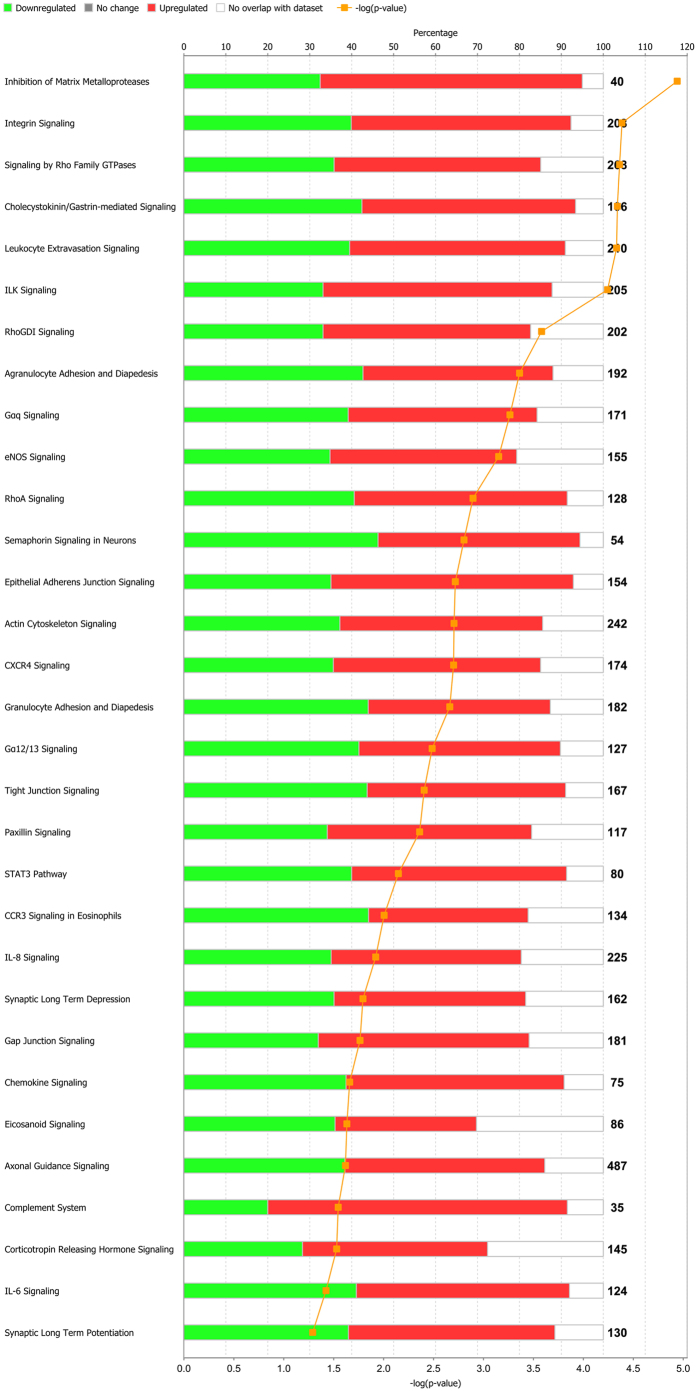
List of pathways sorted by enrichment *P* values, with specification of the percentage of up-regulated and down-regulated genes, when compared with the total number of genes known to participate in the pathways. The orange curve shows the ratio between the number of differentially expressed genes and the total number of genes in these pathways.

Enriched pathways were selected on the basis of their significance levels (−log(*P*-values) ≥1.3) and are listed in Fig. 1 in scoring order (by increasing Fisher’s exact test *P* value right tailed). They can be divided into 5 partially overlapping classes of pathways: cell migration (ILK signaling, integrin signaling, inhibition of matrix metalloproteases, CXCR4 signaling), cell signaling (signaling by Rho family GTPases, RhoGDI signaling, Gαq signaling, eNOS signaling, RhoA signaling, chemokine signaling), neuron signaling (semaphorin signaling in neurons, axonal guidance signaling, synaptic long-term depression/potentiation), immune response (leukocyte extravasion signaling, agranulocyte adhesion and diapedesis, complement system), and actin stress fiber formation and regulation (cholecystokinin/gastrin-mediated signaling).

**Figure 2:**
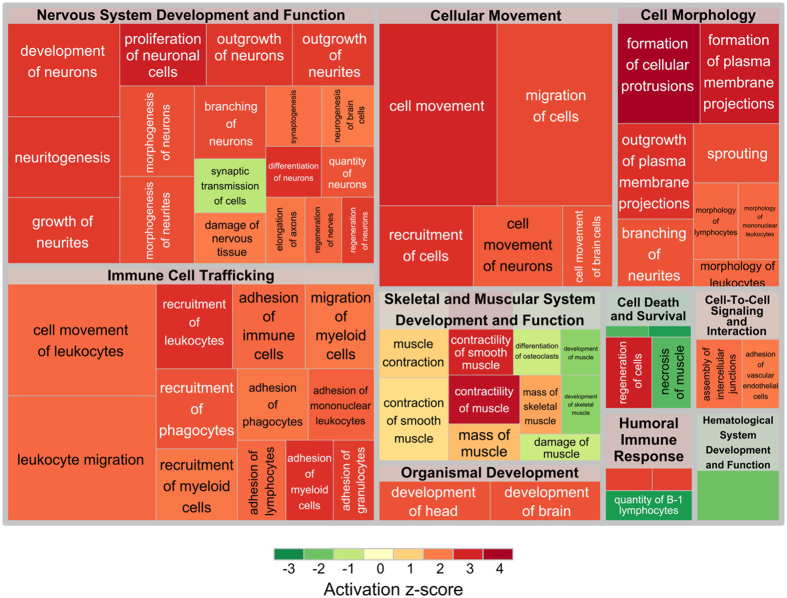
Treemap representing diseases and functions, as calculated by IPA, grouped in macro-processes. Colors indicate the activation z-score of processes: activated processes are colored in red, while inhibited processes are colored in green. Sizes of squares are proportional to −log(p-values). Thus, the greater the size of a square, the more significant its enrichment.

Diseases and Functions analysis was performed by means of Ingenuity Pathway Analysis (IPA). It returned a number of biological functions grouped into categories (macro-processes) (Fig. 2). Categories were ranked by a score that weighted the absolute z-scores of the individual diseases and functions belonging to the categories. The weighted scores were the complement of the inverse −log(P-value) (see Material and Methods). The three top categories resulted: *Nervous System Development and Function, Immune Cell Trafficking* and *Skeletal and Muscular System Development and Function*. Cell Morphology and Cellular Movement Categories were skipped because they are functionally close to the first two categories and, thus, they can be clearly be assimilated to them (Table 3).

Summarizing, these three macro-processes can be grouped into two physiological categories of processes: neuronal/muscular and neuronal/immunity processes. The former was enriched by genes responsible for the increased contraction and contractility of smooth muscle (z-score = 1.455 and z-score = 2.623, respectively; Tables S1 and S2), for the increased damage to the nervous system (z-score = 1.611; Table S3), and for the reduced activity of synaptic transmission (z-score = −1.062; Table S4). On the other hand, the latter comprised genes involved in immune response and nerve regeneration. In particular, we measured increased leukocyte migration, recruitment of phagocytes, and adhesion of immune cells (z-score = 2.474, z-score = 2.416, and z-score = 2.416, respectively; Tables S5, S6 and S7), as well as increased nerve regeneration and synaptogenesis (z-score = 2.200 and z-score = 2.164, respectively; Tables S8 and S9).

### Upstream transcription regulators and functional networks over-representation

**Figure 3:**
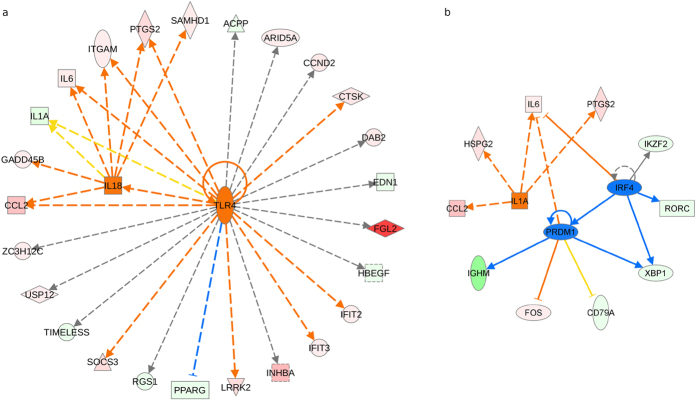
(**a**) Upstream regulators *TLR4* (FC 2.566, z-score = 2.344) and *IL18* (FC −4.559, z-score = 2.025) predicted to have enhanced activity in light of the fold changes in the expression of their target genes. (**b**) Upstream regulators *IL1A* (FC −5.208, z-score = 1.985), *IRF4* (FC −2.188, z-score = −1.969), and *PRDM1* (FC −4.254, z-score = −1.698) with a strong trend toward significance, namely with z-scores close to ±2.

The 180 genes involved in the above-mentioned processes were subjected to upstream regulator analysis, a powerful function of IPA which can, by analyzing linkage to DEGs through coordinated expression, identify potential upstream regulators including transcription factors and any gene or small molecule that have been observed experimentally to affect gene expression^22^. According to the achieved z-scores, *TLR4* (FC 2.566, z-score = 2.344) and *IL18* (FC −4.559, z-score = 2.025) resulted the most significant and activated regulators (Fig. 3a). Relevant z-scores were also obtained for *IL1A* (FC −5.208, z-score = 1.985), predicted as ‘activated’, and *IRF4* (FC −2.188, z-score = −1.969) and *PRDM1* (FC −4.254, z-score = −1.698) (Fig. 3b), predicted as ‘inhibited’.

DEGs were further screened to pinpoint network-eligible molecules. These molecules wired 47 networks (Table S10). Of the two networks exhibiting the highest scores, one over-represented cell morphology, humoral immune response, and cellular movement processes (Figure S2), while the other over-represented organ morphology, skeletal and muscular system development processes, and neurological disease (Figure S3). Both of their scores were 33, implying that there was a 1 in 10^33^ chance of getting a network containing at least the same number of network-eligible molecules when randomly picking 35 molecules (i.e., the maximum possible size of a network) that can be in networks from the Ingenuity Knowledge Base. The former network exhibited a ρ value of 5.25, and the latter a value of 6.01. Hence, both networks were not sparse, thus indicating dense interactions among network-eligible molecules, which may not be due to chance.

## Discussion

Idiopathic achalasia is characterized by the destruction of myenteric neurons synthesizing nitric oxide (NO), responsible for the inhibitory component of esophageal peristalsis and LES relaxation. Pathological examination of affected LES tissue has revealed an inflammatory response with T cell infiltrates surrounding the area of neuronal^23^. However, the trigger for inflammation remains unknown. A recently proposed model suggested that a genetically predisposed individual exposed to an infection mounts a chronic inflammatory response with ensuing neuronal destruction^3^,^18^,^19^.

Analysis of gene expression profiling has been widely used to reveal abnormally expressed genes associated with several diseases at the tissue and cellular levels^24^. This is the first study where genome-wide expression has been investigated in mRNA extracted from the tissue of patients with achalasia, and compared with that of control patients without achalasia. We identified an up-regulated series of mostly DEGs like *TPM2* and *ITGA*, and *EPHA7*, which are involved in smooth muscle contraction. The *TPM2* gene encodes beta-tropomyosin, a member of the actin filament binding protein family, mainly expressed in slow, type 1 muscle fibers. Tropomyosin is a component of the muscle sarcomeric thin filament and plays a crucial role in the calcium-dependent regulation of muscle contraction. Mutations in this gene can alter the expression of other sarcomeric tropomyosin proteins, and cause cap disease and nemaline myopathy^25^, and distal arthrogryposis syndromes^26^. The *ITGA1* gene encodes the alpha 1 subunit of integrin receptors. Integrins are members of a family of heterodimeric cell-surface proteins that mediate cell extracellular matrix and cell–cell interactions; in addition, they couple the cell extracellular matrix to the cytoskeleton (in particular, the microfilaments inside the cell). Integrins mediate signaling events that are essential for stable cell adhesion, spreading, migration, survival, proliferation, and differentiation. The integrin, alpha 1 subunit is involved in the adhesion and dissemination of gastric cancer cells to the peritoneum^27^. The up-regulation of *EPHA7* is notable, as the role of ephrin receptors and their ligands, ephrins, has been largely studied during the development of the nervous system, including axon guidance, axon fasciculation, neural crest cell migration, angiogenesis, and neuronal cell survival during embryonic development^28^.

Genes implicated in innate defense against pathogens, like *CXCL17*, *IGHA1*, and *IGHA2*, were down-regulated in patients with achalasia. The *CXCL17* gene codes for a homeostatic, mucosa-associated chemokine involved in innate immunity that accelerates tumor angiogenesis and progression^29^. A recent study even shows that the CXCL17 peptide exhibits antimicrobial activity potentially through disrupting bacterial membrane^30^. *IGHA1* and *IGHA2* are protein-coding genes that may serve both to defend against local infection and to prevent access of foreign antigens to the immune system.

Pathway enrichment analysis of the sample data was performed to elucidate the biological significance of the differential expression pattern in patients with achalasia and controls. The resulting pathways were grouped into five classes according to both their modulation significance and their presumed relevance for achalasia onset and development. Of interest, when we selected genes involved in pathways and functions highly related to the disease, two critical physiological categories of processes were highlighted: the neuronal/muscular processes and neuronal/immunity organismal responses. The list of functional categories was dominated by inflammation-related categories. There were several categories describing leukocyte migration, recruitment of phagocytes, and adhesion of immune cells. Interestingly, several categories related to the contraction and contractility of smooth muscle, damage to the nervous system, and synaptic transmission activity were broadly highlighted, supporting the notion that these may be important processes in achalasia.

Degenerative alterations and loss of myenteric ganglion cells and nerves associated with an inflammatory infiltrate support the hypothesis that achalasia is the result of a chronic inflammatory process^31^. At the same time, the presence of a lymphocytic infiltrate within the LES, the occurrence of circulating antimyenteric neuronal antibodies as well as the association with antigens of class II major histocompatibility complex^11^ support the existence of immuno-inflammatory mechanisms leading to neuronal cell loss. Patients with achalasia are characterized by significantly higher esophageal lymphocyte infiltration, mainly represented by CD3+CD8+ T cells and IL-1β, IFNγ, and IL-2^32^. Of note in our study, various cytokines and chemokines (IL-6, IL-1RN, IL-33, IL-618, IL-1A, CCL2, CCL24, CXCL12, CXCL13, and CXCL14) were deregulated in the identified processes. Of interest, IL-6 has been shown to play a central role in the neuronal reaction to nerve injury. Suppression of IL-6R by *in vivo* application of anti-IL-6R antibodies led to reduced regenerative effects^33^.

The esophageal wall and LES are innervated by postganglionic neurons, consisting of excitatory neurons releasing acetylcholine and inhibitory neurons that release NO and vasoactive intestinal polypeptide (VIP), resulting in esophageal and LES contraction and relaxation, respectively^34^. The loss of NO and VIP releasing inhibitory neurons, occurring possibly concurrently, results in the clinical consequences seen in patients with achalasia^1^. The etiology of neuronal alterations detected in primary achalasia remains unknown. In cultured neurons, the important role of Ca^2+^, which is a second cellular messenger coupled to neuronal activity, to synaptic plasticity and the regulation of gene expression has been noted^35^. In neurodegenerative disorders, cellular Ca^2+^ regulating systems are compromised, resulting in excitotoxic damage, neuronal degeneration, and forms of programmed cell death^36^. Of interest, calcium channel blockers that inhibit cellular uptake of calcium necessary for contraction, have traditionally been administered to patients with achalasia who are unable to undergo more invasive treatment^37^. We observed a number of sequences involving the calcium signaling pathway (ie, *CACNA1C*, *CACNB2*, *KCNMA1*, *CHRM2*, *CAV1*, and *NCS1*) that were differentially expressed and were able to characterize these genes into possible functions related to achalasia pathogenesis. A better understanding of the cellular and molecular mechanisms that promote or prevent disturbances in cellular Ca^2+^ homeostasis may lead to novel approaches for therapeutic intervention in neurological disorders such as achalasia, Alzheimer’s and Parkinson’s diseases.

Upstream regulator analysis by IPA identified the *TLR4* and *IL18* genes as the top-most predicted activated upstream regulators. A mechanistic network generated for *TLR4* in this analysis (see Fig. 3) shows that it may exert its effects on the observed gene expression by interacting with *IL18* and also through co-activators and other regulatory molecules. TLRs are a family of transmembrane protein receptors that recognize a diverse range of signals on exogenous and endogenous substances considered to be danger signals^38^. *TLR4* recognizes different viruses and bacteria by pathogen-associated molecular patterns and when activated recruits adaptor molecules and kinases, initiating a downstream signaling cascade that culminates in the secretion of proinflammatory cytokines and chemokines (TNF, IL-1α, IL-1β, IL-6, IL-8, IL-10, IL-18, and IL-1A)^39^. These neuroinflammatory mediators are able to activate microglia leading to neuronal excitation or neuronal loss^40^. It is therefore suspected that *TLR4*-mediated glial activation results in the production of NO oxide, oxygen derived free radicals, proteases, adhesion molecules, and proinflammatory cytokines which, when produced in excess, have detrimental effects on neuronal homeostasis and contribute to the development of neurodegenerative conditions such as Alzheimer’s disease^41^. Viruses, such as herpes simplex virus 1 (HSV-1), measles, and human papillomavirus, have been proposed as potential antigens leading to achalasia^42^.

The two top-most predicted inhibited upstream regulators (Fig. 3b) are *IRF4* and *PRDM1. IRF4* is a lymphocyte-restricted transcription factor that is part of a family of DNA-binding proteins critical for the function and homeostasis of mature B and T cells, and mediates signaling in response to a range of cytokines^43^. *IRF4* is closely associated with the human T cell leukemia virus transformation process and is also involved in Epstein-Barr virus-mediated transformation of human B lymphocytes^44^. The gene has a role in the negative feedback regulation of TLR signaling that is central to the activation of innate and adaptive immune systems. The positive regulatory domain zinc finger protein 1 (*PRDM1*) gene plays an essential role in the differentiation of B lymphocytes into plasma cells, a process regulated by *IRF4* by controlling the expression of the *PRDM1* gene in a graded manner^45^. *IRF4* and *BLIMP1* activate the expression of *XBP1*. High levels of *IRF4*, *XBP1*, and *BLIMP1* then jointly promote the differentiation of memory B cells into plasma cells^46^. Interestingly, in our results, *IRF4* and *BLIMP1* are important upstream regulators of genes down-regulated, such as *XBP1*, *IGHM*, *CD79A*, *RORC*, and *IKZF2*, stressing the implications of the immune-inflammation network in achalasia.

In conclusion, these data provide a rigorously characterized expression profile of the whole genome in the esophageal tissue of patients with achalasia. Our findings identify a number of key regulators and pathways of idiopathic achalasia worthy of further study. Genes and pathways that participate in neuronal/muscular processes as well as neuronal/immunity organismal responses were highlighted, and potentially contribute to the onset and development of achalasia. In particular, we pinpoint the role of the *TLR4* and *IL18* genes as major predicted activated upstream regulators. We also present evidence for links between achalasia and calcium signaling. These insights suggest several new avenues for mechanistic research into idiopathic achalasia and potential drug targets.

## Materials and Methods

### Patients and clinical data

LES muscle specimens from 12 patients with achalasia undergoing myotomy were obtained at the time of surgery. Control specimens, consisting of similar muscle samples taken from 5 patients undergoing distal esophagectomy for fundic gastric cancer, were compared with the achalasia biopsies. All patients were recruited at the IRCCS Hospital ‘Casa Sollievo della Sofferenza’, San Giovanni Rotondo, Italy. Achalasia was diagnosed according to esophageal manometry, radiological and endoscopic standard^47^,^48^. Esophageal manometry was carried out using a low compliance perfusion system, as previously described^49^. Clinical data, including demographics, esophageal diameter (evaluated by esophageal barium swallow), basal pressure, symptom score, presence of vigorous (>40 mm Hg) contractions, and type of and response to treatment, were retrieved from medical records. Only one patient presented with a concomitant autoimmune disease (thyroid autoimmunity), and none had an associated esophageal cancer. The study and the experimental protocols were approved by the ethics committee of the IRCCS Hospital ‘Casa Sollievo della Sofferenza’ and were performed in accordance with the approved guidelines. All participants gave written informed consent before study entry.

### Muscle specimens

For each patient with achalasia, a muscle slip measuring approximately 1 cm in length was taken from the proximal region of the LES. The LES was identified intra-operatively using on-table simultaneous endoscopy and laparoscopy. In controls, the muscle biopsy was taken from the upper region of a surgical tumor-free specimen. Then 4 μm-thick optimal cutting temperkature compound embedded fresh tissue sections were cut and routinely stained (hematoxylin and eosin). An expert pathologist assessed the adequacy of the sample ensuring the absence of neoplastic cells.

### RNA isolation

The patient and control specimens were immediately stabilized in liquid nitrogen and kept at −80 °C until isolation of RNA. Total RNA was extracted using Trizol (Invitrogen, Paisley, UK) and the RNeasy Mini Kit (QIAGEN, Hilden, Germany), as directed by the manufacturers. Purified RNA was then quantified using the NanoDropTM 1000 Spectrophotometer (NanoDrop Technologies, Berlin, Germany) and RNA quality was determined by running aliquots on the 2100 Bioanalyzer (Agilent Technologies, Waldbronn, Germany). Samples with a 28S/18S ratio <1.0, RNA integrity number <6.5, or a concentration <30 ng/μL were excluded from microarray experiments.

### Microarray analysis

Gene expression profiling was performed using GeneChip Human Gene 2.0 ST Arrays following the manufacturer’s instructions (Affymetrix, Santa Clara, CA). Briefly, on 100 ng of total RNA, a random priming method was used to generate cDNA from all RNA transcripts present in a sample. The cDNA was fragmented and labeled with biotin using terminal deoxynucleotidyl transferase (TdT) before hybridization in a GeneChip Hybridization Oven 645 (Affymetrix). Following hybridization and post-hybridization washes, the arrays were scanned using the Affymetrix GeneChip Scanner 3000 7G to generate the raw data (CEL file). The quality control steps of the experiment were performed using Expression Console v1.3 (Affymetrix). The messenger RNA expression data have been submitted to ArrayExpress Annotare 2.0 (https://www.ebi.ac.uk/fg/annotare) with the series accession number E-MTAB-3962.

### Data analysis

#### Gene expression microarray analysis

Expression data were analyzed with Partek Genomics Suite 6.6 and R-3.1.2. In particular, quality assessment and signal normalization were performed with Partek. Filtering and statistical analyses were carried out with R. Raw data were log-transformed and quantiles normalized. Probes not mapping to any Entrez gene were removed. In cases where several probe sets mapped to the same gene, the one exhibiting the highest variance was chosen for further analysis. Batch effects were removed by Partek’s batch effect removal algorithm. Sample size was estimated as in Muller *et al*.^50^. Potential effect sizes and sample sizes were simulated from 1.25 to 3.0 by step 0.25, and from 4 to 34 by step 4, respectively. With the significance threshold (alpha) set to 0.01 and the power (1 − beta) to 0.8, a sample size of 17 was guaranteed to capture 90% of genes that changed by 2.5-fold as statistically significant. The exact power value for these thresholds, i.e. p-value<0.05 and |FC|>2.5, and sample sizes for patients with achalasia and control patients was equal to 0.99. Sample distribution among groups was assessed using principal component analysis (PCA). Correction for multiple test was achieved by the Benjamini-Hochberg procedure. Statistical differences in gene expression were assessed by the ANOVA test. The significance threshold was set to 0.05.

#### Functional enrichment analysis

Disease, function, and pathway analyses were conducted using Ingenuity Pathway Analysis (IPA; QIAGEN, Redwood City, CA; www.qiagen.com/ingenuity). The entire procedure was based on the prior calculation of the activation z-scores, which infer the activation states of predicted transcriptional regulators, functions, and pathways. Inference of activating or inhibiting molecules or biological functions is based on confirmation by the literature of the results of our experiments.

Categories of diseases and functions annotations were ranked by a score computed with a weighted sum of the absolute z-scores (*zs*) of the diseases and functions belonging to the categories. Weights were calculated as the complement of the inverse of −log(p-value) (*pv*). For instance, considering the Cellular Movement (CM) process, we grouped the z-scores and p-values of *recruitment of cells* (RC), *cell movement of neurons*, *cell movement of brain cells*, *cell movement and migration of cells* (MC) processes according to the following formula:
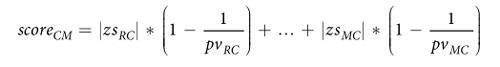


Generally, given the observed differential regulation of a gene, the activation state of an upstream regulator was determined by the regulation direction associated with the relationship from the regulator to the gene. An enrichment score (Fisher’s exact test, *P*-value) was calculated to measure the overlap between observed and predicted regulated gene sets^22^. We considered *P*-values <0.05, z-scores >2 (minimum activation threshold), and z-scores < −2 (minimum inhibition threshold) as significant. The inference of an upstream regulator was based on an expected causal effect, known in the literature and exerted by upstream regulators to targets. The analysis considered all known targets of each putative upstream regulator in the dataset and compares the targets’ actual direction of gene expression change to expectations derived from the literature. The result was a prediction for each upstream regulator. When the direction of change was consistent with the literature, the upstream regulator was predicted to be more active in the experimental sample than in the control. In contrast, when the upstream regulator was inconsistent, it was predicted to be less active.

Finally, gene interactions were checked against the Ingenuity Knowledge Base and interacting genes were identified as network-eligible molecules, which served as *seeds* for generating networks. Networks were scored based on the number of network-eligible molecules they contained. Scores were based on the hypergeometric distribution and were calculated with the right-tailed Fisher’s exact test. The higher the score, the lower the probability of finding the observed number of network-eligible molecules in a given network by chance. Moreover, the higher the interconnection among genes in a network, the higher the probability that the network represents significant biological functions. Density of networks was calculated by the completeness index ρ^51^,^52^. Dense networks have ρ > 1, sparse networks have ρ < 1. The more extreme the value of ρ, the denser or sparser the corresponding network. Technically, networks were represented as mixed graphs, that is they contained a set of undirected edges and a set of directed edges. Edges represented relationships between two molecules. These were undirected if the relationships they represented were not causal, and thus they were ‘affects on’ relationships. Moreover, edges were represented as dashed lines if they were indirect interactions. Orange and blue nodes were predicted to have enhanced or reduced activity, respectively. Orange and blue edges indicated activating and inhibiting action exerted by the issuing node to the target node. Yellow edges represented findings inconsistent with the state of downstream molecules. Gray edges were used when it was not possible to predict the effect of an interaction. Finally, the shape of the node indicated the type of the corresponding molecule, for example, square nodes were cytokines, dashed square nodes were growth factors, triangles were kinases and vertical diamonds are enzymes.

## Additional Information

**How to cite this article**: Palmieri, O. *et al*. Gene expression of muscular and neuronal pathways is cooperatively dysregulated in patients with idiopathic achalasia. *Sci. Rep.* **6**, 31549; doi: 10.1038/srep31549 (2016).

## Supplementary Material

Supplementary Information

## Figures and Tables

**Table 1:** General characteristics of patients with achalasia and controls.

Male/female patients	4/8 (33% male)
Mean age, y	58.5 ± 15.7
Males	55.8 ± 16.3
Females	60.5 ± 16
Age at diagnosis, y	56.1 ± 18.1
LES basal pressure, mm Hg	38.8 ± 14.4
Duration of symptoms before diagnosis, y	
≤1	6
>1	6
Dysphagia, n (%)	12 (100%)
Esophageal regurgitation, n (%)	12 (100%)
Chest pain, n (%)	0
Weight loss	
>5 kg (%)	6 (50%)
<5 kg (%)	6 (50%)
Male/female controls	5/0 (100% M)
Mean age, y	66.6 ± 10

Values are expressed as means ± SD.

LES, lower esophageal sphincter.

**Table 2:** Expression changes in the most dysregulated sequences comparing the resection specimens in achalasia patients and in controls.

Gene symbol	RefSeq	*P* value	Fold change
*EPHA7*	NM_004440	1.76E-08	35.2916
*MSRB3*	NM_001193460	2.44E-08	4.05659
*FERMT2*	NM_006832	5.66E-08	4.98674
*SOBP*	NM_018013	1.41E-07	3.55806
*CAMK2G*	NM_172171	1.70E-07	2.95387
*TPM2*	ENST00000378292	4.05E-07	5.33501
*NRP2*	ENST00000360409	6.33E-07	6.56911
*MIR100HG*	NR_024430	6.96E-07	3.67597
*ITGA1*	ENST00000282588	7.09E-07	4.12193
*DGKG*	NM_001346	9.47E-07	7.27657
*NEGR1*	ENST00000357731	1.06E-06	6.67641
*DSTN*	NM_001011546	1.06E-06	4.17867
*SESTD1*	ENST00000428443	1.41E-06	3.62844
*EFEMP2*	NM_016938	1.51E-06	4.2466
*ADCY5*	NM_183357	2.52E-06	8.12951
*ILK*	NM_001014795	3.93E-06	2.98349
*PDLIM3*	NM_014476	4.68E-06	7.96164
*CLIC4*	NM_013943	5.41E-06	3.77628
*ITGB1BP2*	ENST00000373829	7.60E-06	4.17973
*FAM184A*	NM_024581	8.00E-06	2.7413
*CXCL17*	NM_198477	2.86E-12	−35.145
*CYP2C18*	NM_000772	1.22E-09	−62.2585
*LIPH*	NM_139248	2.46E-09	−35.769
*ESRP1*	NM_017697	2.95E-09	−28.4784
*SH3RF2*	NM_152550	8.29E-09	−9.73303
*IGHA2*	BX640625	1.16E-08	−32.3116
*CDH1*	NM_004360	2.02E-08	−41.6101
*SPINT2*	NM_021102	3.20E-08	−12.3322
*ESRP2*	NM_024939	4.03E-08	−7.40189
*GRHL2*	NM_024915	4.50E-08	−9.60162
*PRSS8*	NM_002773	5.31E-08	−9.27125
*IGHA1*	AK125238	6.92E-08	−26.6764
*SCNN1A*	NM_001159576	7.61E-08	−6.76352
*OCLN*	NM_002538	8.84E-08	−23.3503
*TJP3*	NM_001267560	1.26E-07	−7.80519
*TFCP2L1*	NM_014553	2.48E-07	−9.38368
*CAMSAP3*	NM_001080429	5.15E-07	−3.78451
*RIPK4*	NM_020639	5.96E-07	−6.65578
*GPT2*	ENST00000440783	6.42E-07	−6.38529
*IRF6*	NM_006147	6.58E-07	−10.8859

RefSeq, NCBI Reference Sequence Database.

**Table 3:** Macro-processes with their associated scores.

Category	Weighted score
Nervous System Development and Function	31.7648
Immune Cell Trafficking	22.4096
Cell Morphology	16.1558
Cellular Movement	11.4097
Skeletal and Muscular System Development and Function	10.4342
Cell Death and Survival	5.7306
Humoral Immune Response	5.0347
Cell-To-Cell Signaling and Interaction	4.0357
Organismal Development	4.0192
Hematological System Development and Function	1.7531

Scores were calculated as the weighted sum of the z-scores of the constituting processes, where weights are the complement of the inverse of −log(P-value).
