# Supplementary material for: Gene expression of muscular and neuronal pathways is cooperatively dysregulated in patients with idiopathic achalasia
Source: Sci Rep. 2016 Aug 11;6:31549. doi: 10.1038/srep31549 (PMC4980661; doi:10.1038/srep31549)
Supplement: Supplementary Information [file srep31549-s1.pdf]

Gene expression of muscular and neuronal pathways is cooperatively dysregulated in patients with idiopathic achalasia

Orazio Palmieri[1], Tommaso Mazza[2], Antonio Merla[1], Caterina Fusilli[2], Antonello Cuttitta[3], Giuseppina Martino[1], Tiziana Latiano[1], Giuseppe Corritore[1], Fabrizio Bossa[1], Orazio Palumbo[4], Lucia Anna Muscarella[5], Massimo Carella[4] , Paolo Graziano[6], Angelo Andriulli[1], Anna Latiano[1*]

1. Division of Gastroenterology, IRCCS ‘Casa Sollievo della Sofferenza’, San Giovanni Rotondo, Foggia, Italy; 2. Bioinformatics Unit, IRCCS ‘Casa Sollievo della Sofferenza’, San Giovanni Rotondo, Foggia, Italy; 3. Unit of General Surgery 2nd and Thoracic Surgery, IRCCS ‘Casa Sollievo della Sofferenza’, San Giovanni Rotondo, Foggia, Italy; 4. Medical Genetics Service, IRCCS ‘Casa Sollievo della Sofferenza’, San Giovanni Rotondo, Foggia, Italy; 5. Laboratory of Oncology, IRCCS ‘Casa Sollievo della Sofferenza’, San Giovanni Rotondo, Foggia, Italy: 6. Pathology Unit, IRCCS ‘Casa Sollievo della Sofferenza’, San Giovanni Rotondo, Foggia, Italy

***Correspondence to**

Anna Latiano BSc, PhD, Division of Gastroenterology, IRCCS ‘Casa Sollievo della Sofferenza’ Hospital, San Giovanni Rotondo, 71013, Foggia, Italy. E-mail: a.latiano@operapadrepio.it. Telephone n.: +39 0882-416281; fax n.: +39 0882-416271.

Table S1. Differentially expressed genes involved in the smooth muscle contraction pathway (z-score = 1.455)

RefSeq	Genes in dataset	Prediction (based on expression direction)	Fold change	Findings
NM_001006630	*CHRM2*	Affected	9.241	Affects (3)
NM_001161353	*KCNMA1*	Affected	8.739	Affects (2)
NM_001299	*CNN1*	Affected	8.268	Affects (1)
NM_002474	*MYH11*	Increased	7.704	Increases (3)
NM_001146312	*MYOCD*	Increased	6.758	Increases (1)
NM_000064	*C3*	Increased	5.057	Increases (2)
NM_000702	*ATP1A2*	Affected	5.003	Affects (1)
NM_199460	*CACNA1C*	Affected	4.715	Affects (2)
NM_001141945	*ACTA2*	Affected	4.260	Affects (1)
NM_000963	*PTGS2*	Increased	3.762	Increases (1)
NM_001172895	*CAV1*	Affected	3.686	Affects (3)
NM_003131	*SRF*	Increased	3.101	Increases (1)
NM_005406	*ROCK1*	Affected	2.755	Affects (1)
NM_000962	*PTGS1*	Increased	2.544	Increases (1)
NM_001002029	*C4A/C4B*	Increased	2.500	Increases (1)
ENST00000315872	*ROCK2*	Affected	2.046	Affects (1)
NM_006681	*NMU*	Decreased	–2.017	Increases (2)
NM_001955	*EDN1*	Decreased	–2.089	Increases (3)

Fold change in expression and the number of scientific papers reporting the predictions are reported in the last two columns.

Table S2. Differentially expressed genes involved in the smooth muscle contractility pathway (z-score = 2.623)

RefSeq	Genes in dataset	Prediction (based on expression direction)	Fold change	Findings
NM_001128205	*SULF1*	Increased	4.021	Increases (2)
NM_001098579	*MRVI1*	Increased	4.248	Increases (2)
NM_001172895	*CAV1*	Increased	3.686	Increases (3)
NM_002474	*MYH11*	Increased	7.704	Increases (3)
NM_001006630	*CHRM2*	Increased	9.241	Increases (4)
NM_001098512	*PRKG1*	Increased	4.769	Increases (1)
NM_198596	*SULF2*	Increased	2.266	Increases (2)

Fold change in expression and the number of scientific papers reporting the predictions are reported in the last two columns.

Table S3. Differentially expressed genes involved in the damage to nervous tissue pathway (z-score = 1.611)

RefSeq	Genes in dataset	Prediction (based on expression direction)	Fold change	Findings
NM_002006	*FGF2*	Decreased	8.752	Decreases (3)
ENST00000361475	*TGM2*	Decreased	5.507	Decreases (7)
NM_000963	*PTGS2*	Increased	3.762	Increases (1)
ENST00000520164	*EBF2*	Affected	3.594	Affects (1)
NM_001111283	*IGF1*	Decreased	3.566	Decreases (23)
NM_001168272	*ITPR1*	Increased	3.540	Increases (1)
NM_031850	*AGTR1*	Increased	2.581	Increases (1)
ENST00000404625	*IL6*	Increased	2.531	Increases (6)
NM_000311	*PRNP*	Increased	2.415	Increases (3)
ENST00000316403	*CLU*	Affected	2.011	Affects (2)
NM_001145412	*NFE2L2*	Increased	–2.139	Decreases (2)
NM_004836	*EIF2AK3*	Increased	–2.604	Decreases (1)
ENST00000278836	*MYRF*	Increased	–3.038	Decreases (2)

ENST00000397026	*PPARG*	Increased	–3.583	Decreases (5)
NM_175617	MT1E	Increased	–4.288	Decreases (1)
ENST00000216492	CHGA	Affected	–4.751	Affects (1)
ENST00000296677	F2RL1	Decreased	–5.020	Increases (1)
NM_005951	MT1H	Increased	–10.206	Decreases (1)

Fold change in expression and the number of scientific papers reporting the predictions are reported in the last two columns.

Table S4. Differentially expressed genes involved in the synaptic transmission of cells pathway (z-score = –1.062)

RefSeq	Genes in dataset	Prediction (based on expression direction)	Fold change	Findings
NM_005314	*GRPR*	Affected	18.122	Affects (1)
ENST00000264381	*BCHE*	Decreased	16.378	Decreases (1)
NM_001006630	*CHRM2*	Increased	9.241	Increases (2)
NM_014286	*NCS1*	Increased	9.111	Increases (1)
NM_001161353	*KCNMA1*	Affected	8.739	Affects (2)
NM_201596	*CACNB2*	Affected	7.862	Affects (1)
NM_001165037	*GABRA5*	Affected	6.850	Affects (1)
NM_000109	*DMD*	Affected	6.824	Affects (1)
NM_001390	*DTNA*	Affected	5.053	Affects (1)
NM_015669	*PCDHB5*	Affected	4.979	Affects (1)
NM_199460	*CACNA1C*	Affected	4.715	Affects (1)
NM_001796	*CDH8*	Affected	3.811	Affects (1)
NM_021032	*FGF12*	Affected	3.755	Affects (1)
NM_015678	*NBEA*	Affected	3.671	Affects (1)
NM_001111283	*IGF1*	Increased	3.566	Increases (1)
NM_000304	*PMP22*	Affected	3.420	Affects (1)
NM_020957	*PCDHB16*	Affected	2.989	Affects (1)
ENST00000334082	*HOMER1*	Affected	2.787	Affects (3)
NM_001256012	*MYH10*	Affected	2.623	Affects (1)
NM_002088	*GRIK5*	Affected	2.510	Affects (1)
NM_000311	*PRNP*	Decreased	2.415	Decreases (1)
NM_000909	*NPY1R*	Affected	2.342	Affects (1)
NM_002522	*NPTX1*	Affected	2.231	Affects (1)
NM_014330	*PPP1R15A*	Increased	2.219	Increases (1)
NM_006154	*NEDD4*	Decreased	2.167	Decreases (2)
NM_001166160	*PPP1R9A*	Affected	2.138	Affects (1)
NM_014905	*GLS*	Affected	2.119	Affects (1)
NM_001135659	*NRXN1*	Affected	2.017	Affects (1)
NM_006681	*NMU*	Decreased	–2.017	Increases (1)
NM_001048	*SST*	Affected	–2.108	Affects (1)
NM_004762	*CYTH1*	Decreased	–2.128	Increases (1)
NM_176875	*CCKBR*	Decreased	–2.512	Increases (1)
NM_004170	*SLC1A1*	Affected	–2.726	Affects (1)
NM_001049	*SSTR1*	Affected	–2.814	Affects (2)
NM_004999	*MYO6*	Affected	–3.354	Affects (1)
NM_000379	*XDH*	Decreased	–3.627	Increases (3)

Fold change in expression and the number of scientific papers reporting the predictions are reported in the last two columns.

Table S5. Differentially expressed genes involved in the leukocyte migration pathway (z-score = 2.474)

RefSeq	Genes in dataset	Prediction (based on expression direction)	Fold change	Findings
NM_138576	*BCL11B*	Increased	–2.345	Decreases (3)
NM_001048	*SST*	Increased	–2.108	Decreases (1)
NM_001920	*DCN*	Increased	3.792	Increases (1)
NM_001554	*CYR61*	Increased	2.224	Increases (3)
NM_002922	*RGS1*	Increased	–2.448	Decreases (4)
NM_000929	*PLA2G5*	Increased	3.492	Increases (5)
ENST00000404625	*IL6*	Increased	2.531	Increases (12)
NM_003248	*THBS4*	Increased	5.623	Increases (1)
ENST00000466675	*RARRES2*	Increased	2.054	Increases (15)
NM_002704	*PPBP*	Increased	5.727	Increases (14)
NM_001957	*EDNRA*	Increased	3.166	Increases (1)
NM_000601	*HGF*	Increased	2.103	Increases (5)
NM_006500	*MCAM*	Increased	3.846	Increases (3)
NM_001198	*PRDM1*	Increased	–4.254	Decreases (1)
NM_001078	*VCAM1*	Increased	2.034	Increases (40)
NM_002977	*SCN9A*	Increased	2.485	Increases (1)
ENST00000435819	*CD36*	Increased	2.434	Increases (3)
NM_000899	*KITLG*	Increased	2.754	Increases (12)
NM_000311	*PRNP*	Increased	2.415	Increases (3)
ENST00000272928	*ACKR3*	Increased	4.365	Increases (2)
ENST00000260356	*THBS1*	Increased	3.439	Increases (17)
NM_000212	*ITGB3*	Increased	3.056	Increases (4)
NM_001114753	*ENG*	Increased	2.164	Increases (4)
NM_001736	*C5AR1*	Increased	2.271	Increases (11)
NM_001002029	*C4A/C4B*	Increased	2.500	Increases (1)
NM_004951	*GPR183*	Increased	2.683	Increases (2)
ENST00000278836	*MYRF*	Increased	–3.038	Decreases (4)
ENST00000367976	*CTGF*	Increased	2.124	Increases (2)
NM_000963	*PTGS2*	Increased	3.762	Increases (6)
NM_004621	*TRPC6*	Increased	6.312	Increases (4)
NM_002644	*PIGR*	Increased	–10.606	Decreases (2)
ENST00000537021	*PIP5K1C*	Increased	2.311	Increases (5)
NM_004530	*MMP2*	Increased	2.938	Increases (4)
NM_173842	*IL1RN*	Increased	–6.493	Decreases (6)
NM_000064	*C3*	Increased	5.057	Increases (15)
NM_012431	*SEMA3E*	Increased	4.295	Increases (1)
ENST00000264870	*F13A1*	Increased	2.793	Increases (1)
NM_181523	*PIK3R1*	Increased	2.636	Increases (6)
ENST00000231751	*LTF*	Increased	–4.517	Decreases (4)
NM_003734	*AOC3*	Increased	9.280	Increases (18)
ENST00000398117	*BCL2*	Increased	2.162	Increases (1)
ENST00000361475	*TGM2*	Increased	5.507	Increases (9)
NM_002615	*SERPINF1*	Increased	3.982	Increases (1)
ENST00000266718	*LUM*	Increased	3.916	Increases (9)
NM_000450	*SELE*	Increased	5.496	Increases (57)
NM_020988	*GNAO1*	Increased	2.777	Increases (2)
NM_001145808	*ITGAM*	Increased	2.076	Increases (23)
NM_000906	*NPR1*	Increased	2.220	Increases (1)
NM_006919	*SERPINB3*	Increased	–9.571	Decreases (1)
NM_199168	*CXCL12*	Increased	3.052	Increases (256)

ENST00000297450	*ANGPT1*	Increased	8.781	Increases (2)
ENST00000264741	*ITGA9*	Increased	4.306	Increases (1)
NM_003247	*THBS2*	Increased	2.976	Increases (5)
NM_015055	*SWAP70*	Increased	2.160	Increases (9)
ENST00000373304	*CYSLTR1*	Increased	13.666	Increases (3)
AK130813	*IGHG1*	Increased	–8.159	Decreases (2)
ENST00000263967	*PIK3CA*	Increased	2.205	Increases (1)
NM_006289	*TLN1*	Increased	4.586	Increases (3)
NM_005406	*ROCK1*	Increased	2.755	Increases (8)
NM_002982	*CCL2*	Increased	5.557	Increases (212)
ENST00000242208	*INHBA*	Increased	6.216	Increases (1)
NM_003064	*SLPI*	Increased	–3.388	Decreases (6)
ENST00000354785	*FN1*	Increased	6.598	Increases (22)
NM_001911	*CTSG*	Increased	2.705	Increases (15)
NM_003266	*TLR4*	Increased	2.566	Increases (25)
ENST00000315872	*ROCK2*	Increased	2.046	Increases (1)
NM_001145657	*RAP1GAP*	Increased	–3.928	Decreases (4)
NM_001204286	*MUC1*	Increased	–14.648	Decreases (1)
NM_001031680	*RUNX3*	Increased	–2.236	Decreases (3)
NM_032801	*JAM3*	Increased	4.453	Increases (7)
NM_003080	*SMPD2*	Increased	–2.356	Decreases (1)
NM_001456	*FLNA*	Increased	8.187	Increases (1)
NM_024164	*TPSAB1/TPSB2*	Increased	2.846	Increases (2)
NM_033439	*IL33*	Increased	3.473	Increases (8)
ENST00000391555	*HSPA1A/HSPA1B*	Increased	4.517	Increases (2)
NM_178155	*FUT8*	Increased	–2.396	Decreases (1)
NM_198578	*LRRK2*	Increased	3.595	Increases (1)
NM_001135597	*CCDC88A*	Increased	2.079	Increases (1)
NM_001202429	*ASB2*	Increased	2.282	Increases (1)
NM_177551	*HCAR2*	Increased	–2.059	Decreases (1)
ENST00000353479	*COL17A1*	Increased	–2.640	Decreases (1)
NM_006147	*IRF6*	Decreased	–10.886	Increases (2)
ENST00000368722	*S100A7*	Decreased	–3.175	Increases (8)
NM_005739	*RASGRP1*	Decreased	–2.632	Increases (4)
ENST00000300177	*GREM1*	Decreased	3.513	Decreases (2)
NM_001955	*EDN1*	Decreased	–2.089	Increases (3)
NM_002421	*MMP1*	Decreased	–2.356	Increases (1)
NM_004887	*CXCL14*	Decreased	–4.303	Increases (3)
NM_003254	*TIMP1*	Decreased	2.183	Decreases (3)
NM_001006946	*SDC1*	Decreased	–3.536	Increases (3)
ENST00000222902	*CCL24*	Decreased	–2.161	Increases (13)
NM_006419	*CXCL13*	Decreased	–2.322	Increases (42)
NM_004288	*CYTIP*	Decreased	–2.925	Increases (3)
NM_012092	*ICOS*	Decreased	–2.278	Increases (9)
NM_005060	*RORC*	Decreased	–2.846	Increases (1)
NM_004925	*AQP3*	Decreased	–4.133	Increases (7)
NM_000362	*TIMP3*	Decreased	3.079	Decreases (2)
NM_001562	*IL18*	Decreased	–4.559	Increases (13)
NM_001490	*GCNT1*	Decreased	–5.136	Increases (11)
NM_003102	*SOD3*	Decreased	6.477	Decreases (1)
NM_002843	*PTPRJ*	Decreased	–2.145	Increases (1)
NM_005219	*DIAPH1*	Decreased	–2.679	Increases (6)
NM_002538	*OCLN*	Decreased	–23.350	Increases (2)

NM_003955	*SOCS3*	Decreased	3.685	Decreases (3)
ENST00000504154	*SLIT2*	Decreased	8.499	Decreases (8)
NM_006113	*VAV3*	Decreased	–2.532	Increases (1)
NM_176072	*P2RY2*	Decreased	–3.402	Increases (2)
ENST00000250018	*TPH1*	Decreased	–4.275	Increases (3)
NM_001174167	*SYK*	Decreased	–2.027	Increases (2)
NM_001910	*CTSE*	Decreased	–15.113	Increases (2)
NM_000527	*LDLR*	Decreased	–2.143	Increases (6)
ENST00000369478	*CD2*	Decreased	–2.398	Increases (2)
NM_003253	*TIAM1*	Decreased	–2.094	Increases (1)
NM_005564	*LCN2*	Decreased	–5.985	Increases (3)
NM_001002857	*ANXA2*	Decreased	–2.347	Increases (2)
NM_006080	*SEMA3A*	Decreased	8.997	Decreases (4)
NM_007329	*DMBT1*	Decreased	–2.968	Increases (1)
NM_002426	*MMP12*	Decreased	–2.505	Increases (2)
NM_001993	*F3*	Decreased	–3.002	Increases (3)
ENST00000296677	*F2RL1*	Decreased	–5.020	Increases (6)
NM_002305	*LGALS1*	Decreased	3.121	Decreases (7)
ENST00000297439	*DEFB1*	Decreased	–3.610	Increases (24)
NM_001098512	*PRKG1*	Decreased	4.769	Decreases (3)
NM_003680	*YARS*	Decreased	–2.326	Increases (2)
NM_016946	*F11R*	Decreased	–5.286	Increases (11)
NM_003255	*TIMP2*	Decreased	2.829	Decreases (2)
ENST00000397026	*PPARG*	Decreased	–3.583	Increases (6)
ENST00000380739	*SERPINB1*	Decreased	–3.585	Increases (1)
NM_000379	*XDH*	Decreased	–3.627	Increases (1)
ENST00000368702	*S100A14*	Decreased	–9.354	Increases (2)
NM_020156	*C1GALT1*	Decreased	–2.026	Increases (3)
NM_001204	*BMPR2*	Decreased	2.195	Decreases (5)
NM_004329	*BMPR1A*	Decreased	3.160	Decreases (1)
NM_005761	*PLXNC1*	Decreased	2.094	Decreases (1)
NM_007309	*DIAPH2*	Affected	2.087	Affects (2)
NM_003379	*EZR*	Affected	–2.551	Affects (2)
NM_002737	*PRKCA*	Affected	2.320	Affects (1)
NM_001773	*CD34*	Affected	4.451	Affects (1)
ENST00000282588	*ITGA1*	Affected	4.122	Affects (3)
NM_004360	*CDH1*	Affected	–41.610	Affects (1)
NM_001097579	*GPR34*	Affected	2.508	Affects (1)
NM_001172895	*CAV1*	Affected	3.686	Affects (2)
NM_001207066	*CXADR*	Affected	–11.706	Affects (1)
ENST00000531198	*CRYAB*	Affected	4.493	Affects (2)
NM_001145412	*NFE2L2*	Affected	–2.139	Affects (1)
NM_001014796	*DDR2*	Affected	5.827	Affects (1)
NM_000575	*IL1A*	Affected	–5.208	Affects (2)
NM_001845	*COL4A1*	Affected	3.224	Affects (2)
ENST00000316403	*CLU*	Affected	2.011	Affects (1)
ENST00000410009	*CD207*	Affected	–2.889	Affects (1)
ENST00000377211	*EDNRB*	Affected	2.378	Affects (1)
NM_001127197	*ELF4*	Affected	–2.144	Affects (1)
NM_001014795	*ILK*	Affected	2.983	Affects (1)
NM_001135599	*TGFB2*	Affected	3.520	Affects (1)
NM_001197293	*DPYSL2*	Affected	2.369	Affects (2)

Fold change in expression and the number of scientific papers reporting the predictions are reported in the last two columns.

Table S6. Differentially expressed genes involved in the recruitment of phagocytes pathway (z-score = 2.416)

RefSeq	Genes in dataset	Prediction (based on expression direction)	Fold change	Findings
NM_001554	*CYR61*	Increased	2.224	Increases (1)
ENST00000404625	*IL6*	Increased	2.531	Increases (2)
NM_001078	*VCAM1*	Increased	2.034	Increases (4)
ENST00000435819	*CD36*	Increased	2.434	Increases (2)
ENST00000260356	*THBS1*	Increased	3.439	Increases (2)
NM_001736	*C5AR1*	Increased	2.271	Increases (8)
NM_004621	*TRPC6*	Increased	6.312	Increases (1)
ENST00000537021	*PIP5K1C*	Increased	2.311	Increases (3)
NM_004530	*MMP2*	Increased	2.938	Increases (1)
NM_173842	*IL1RN*	Increased	–6.493	Decreases (3)
ENST00000264870	*F13A1*	Increased	2.793	Increases (1)
ENST00000231751	*LTF*	Increased	–4.517	Decreases (1)
NM_001172895	*CAV1*	Increased	3.686	Increases (1)
NM_000450	*SELE*	Increased	5.496	Increases (5)
NM_001145808	*ITGAM*	Increased	2.076	Increases (3)
NM_199168	*CXCL12*	Increased	3.052	Increases (3)
NM_003247	*THBS2*	Increased	2.976	Increases (1)
ENST00000296677	*F2RL1*	Increased	–5.020	Decreases (1)
NM_005406	*ROCK1*	Increased	2.755	Increases (1)
NM_002982	*CCL2*	Increased	5.557	Increases (31)
NM_003064	*SLPI*	Increased	–3.388	Decreases (4)
NM_001911	*CTSG*	Increased	2.705	Increases (2)
NM_003266	*TLR4*	Increased	2.566	Increases (9)
NM_001204286	*MUC1*	Increased	–14.648	Decreases (1)
NM_033439	*IL33*	Increased	3.473	Increases (2)
ENST00000391555	*HSPA1A/HSPA1B*	Increased	4.517	Increases (2)
NM_178155	*FUT8*	Increased	–2.396	Decreases (1)
NM_177551	*HCAR2*	Increased	–2.059	Decreases (1)
ENST00000353479	*COL17A1*	Increased	–2.640	Decreases (1)
NM_001955	*EDN1*	Decreased	–2.089	Increases (1)
ENST00000466675	*RARRES2*	Decreased	2.054	Decreases (2)
NM_002421	*MMP1*	Decreased	–2.356	Increases (1)
NM_001562	*IL18*	Decreased	–4.559	Increases (5)
NM_001490	*GCNT1*	Decreased	–5.136	Increases (1)
NM_000575	*IL1A*	Decreased	–5.208	Increases (1)
ENST00000504154	*SLIT2*	Decreased	8.499	Decreases (1)
NM_006113	*VAV3*	Decreased	–2.532	Increases (1)
NM_176072	*P2RY2*	Decreased	–3.402	Increases (2)
ENST00000531198	*CRYAB*	Decreased	4.493	Decreases (1)
NM_005564	*LCN2*	Decreased	–5.985	Increases (1)
NM_001993	*F3*	Decreased	–3.002	Increases (1)
ENST00000397026	*PPARG*	Decreased	–3.583	Increases (4)
NM_000379	*XDH*	Decreased	–3.627	Increases (1)
NM_001145412	*NFE2L2*	Affected	–2.139	Affects (1)
ENST00000316403	*CLU*	Affected	2.011	Affects (1)
NM_006147	*IRF6*	Affected	–10.886	Affects (1)

Fold change in expression and the number of scientific papers reporting the predictions are reported in the last two columns.

Table S7. Differentially expressed genes involved in the adhesion of immune cells pathway (z-score = 2.461)

RefSeq	Genes in dataset	Prediction (based on expression direction)	Fold change	Findings
NM_002922	*RGS1*	Increased	–2.448	Decreases (1)
NM_002438	*MRC1*	Increased	2.991	Increases (1)
ENST00000404625	*IL6*	Increased	2.531	Increases (4)
NM_002704	*PPBP*	Increased	5.727	Increases (6)
NM_000601	*HGF*	Increased	2.103	Increases (1)
NM_006500	*MCAM*	Increased	3.846	Increases (1)
NM_001078	*VCAM1*	Increased	2.034	Increases (36)
NM_001144996	*ITGA7*	Increased	3.397	Increases (1)
NM_002332	*LRP1*	Increased	2.693	Increases (2)
NM_000899	*KITLG*	Increased	2.754	Increases (4)
NM_012092	*ICOS*	Increased	–2.278	Decreases (4)
ENST00000260356	*THBS1*	Increased	3.439	Increases (4)
NM_000212	*ITGB3*	Increased	3.056	Increases (2)
ENST00000316403	*CLU*	Increased	2.011	Increases (1)
NM_000963	*PTGS2*	Increased	3.762	Increases (3)
NM_000064	*C3*	Increased	5.057	Increases (2)
ENST00000231751	*LTF*	Increased	–4.517	Decreases (3)
NM_003734	*AOC3*	Increased	9.280	Increases (7)
NM_000450	*SELE*	Increased	5.496	Increases (22)
NM_001145808	*ITGAM*	Increased	2.076	Increases (44)
NM_199168	*CXCL12*	Increased	3.052	Increases (29)
NM_003247	*THBS2*	Increased	2.976	Increases (1)
NM_015055	*SWAP70*	Increased	2.160	Increases (4)
NM_001773	*CD34*	Increased	4.451	Increases (2)
ENST00000263967	*PIK3CA*	Increased	2.205	Increases (1)
NM_006289	*TLN1*	Increased	4.586	Increases (1)
NM_002982	*CCL2*	Increased	5.557	Increases (6)
ENST00000354785	*FN1*	Increased	6.598	Increases (14)
NM_003266	*TLR4*	Increased	2.566	Increases (5)
ENST00000397026	*PPARG*	Increased	–3.583	Decreases (1)
NM_078481	*CD97*	Increased	2.524	Increases (6)
NM_001145657	*RAP1GAP*	Increased	–3.928	Decreases (3)
NM_002444	*MSN*	Increased	2.780	Increases (2)
NM_032801	*JAM3*	Increased	4.453	Increases (3)
NM_033439	*IL33*	Increased	3.473	Increases (2)
NM_005739	*RASGRP1*	Decreased	–2.632	Increases (3)
NM_001920	*DCN*	Decreased	3.792	Decreases (4)
NM_000575	*IL1A*	Decreased	–5.208	Increases (4)
NM_005219	*DIAPH1*	Decreased	–2.679	Increases (1)
ENST00000537021	*PIP5K1C*	Decreased	2.311	Decreases (1)
NM_006113	*VAV3*	Decreased	–2.532	Increases (2)
ENST00000250018	*TPH1*	Decreased	–4.275	Increases (1)
ENST00000369478	*CD2*	Decreased	–2.398	Increases (4)
NM_004360	*CDH1*	Decreased	–41.610	Increases (2)
ENST00000297450	*ANGPT1*	Decreased	8.781	Decreases (3)
NM_005406	*ROCK1*	Decreased	2.755	Decreases (5)
NM_016946	*F11R*	Decreased	–5.286	Increases (2)
ENST00000265769	*ADAM28*	Decreased	–9.698	Increases (1)
ENST00000318602	*A2M*	Decreased	2.301	Decreases (1)
NM_000820	*GAS6*	Decreased	2.105	Decreases (5)

NM_000697	*ALOX12*	Decreased	–3.203	Increases (1)
NM_000062	*SERPING1*	Decreased	4.031	Decreases (5)
NM_005761	*PLXNC1*	Decreased	2.094	Decreases (1)
ENST00000314134	*SLC35C1*	Affected	–2.058	Affects (1)
NM_001911	*CTSG*	Affected	2.705	Affects (4)
NM_003379	*EZR*	Affected	–2.551	Affects (1)
NM_001993	*F3*	Affected	–3.002	Affects (3)
NM_021913	*AXL*	Affected	4.242	Affects (2)
NM_002522	*NPTX1*	Affected	2.231	Affects (1)
NM_001174167	*SYK*	Affected	–2.027	Affects (1)
NM_006419	*CXCL13*	Affected	–2.322	Affects (1)
ENST00000435819	*CD36*	Affected	2.434	Affects (1)

Fold change in expression and the number of scientific papers reporting the predictions are reported in the last two columns.

Table S8. Differentially expressed genes involved in the regeneration of nerves pathway (z-score = 2.200)

RefSeq	Genes in dataset	Prediction (based on expression direction)	Fold change	Findings
NM_002006	*FGF2*	Increased	8.752	Increases (1)
NM_173842	*IL1RN*	Increased	–6.493	Decreases (1)
ENST00000282588	*ITGA1*	Increased	4.122	Increases (1)
NM_199168	*CXCL12*	Increased	3.052	Increases (1)
NM_001111283	*IGF1*	Increased	3.566	Increases (3)
ENST00000316403	*CLU*	Affected	2.011	Affects (1)

Fold change in expression and the number of scientific papers reporting the predictions are reported in the last two columns.

Table S9. Differentially expressed genes involved in the synaptogenesis pathway (z-score = 2.164)

RefSeq	Genes in dataset	Prediction (based on expression direction)	Fold change	Findings
NM_001135659	*NRXN1*	Increased	2.017	Increases (5)
NM_001128310	*SPARCL1*	Increased	5.453	Increases (1)
ENST00000260356	*THBS1*	Increased	3.439	Increases (1)
NM_000212	*ITGB3*	Increased	3.056	Increases (1)
NM_001242607	*NCAM1*	Increased	2.277	Increases (3)
NM_001172895	*CAV1*	Increased	3.686	Increases (1)
NM_003247	*THBS2*	Increased	2.976	Increases (1)
NM_001111283	*IGF1*	Increased	3.566	Increases (2)
ENST00000457714	*NLGN1*	Increased	3.163	Increases (3)
NM_004734	*DCLK1*	Decreased	3.488	Decreases (1)
NM_144505	*KLK8*	Decreased	–2.869	Increases (1)
NM_020957	*PCDHB16*	Affected	2.989	Affects (1)
NM_015669	*PCDHB5*	Affected	4.979	Affects (1)
NM_014987	*IGSF9B*	Affected	3.146	Affects (1)
NM_002923	*RGS2*	Affected	4.092	Affects (1)
NM_001843	*CNTN1*	Affected	15.193	Affects (1)
NM_004360	*CDH1*	Affected	–41.610	Affects (1)
NM_004999	*MYO6*	Affected	–3.354	Affects (1)
NM_016588	*NRN1*	Affected	3.196	Affects (2)
NM_022740	*HIPK2*	Affected	–2.121	Affects (3)
NM_005233	*EPHA3*	Affected	4.199	Affects (1)

Fold change in expression and the number of scientific papers reporting the predictions are reported in the last two columns.

Table S10: regulatory networks explaining the gene expression changes in the dataset.

Molecules in Network	Score	Focus Molecules	Top Diseases and Functions
ACPP,ARID5A,BLNK,CCND2,CD79A,CMA1,CPA3,DAB2,DDR1,EDN1,EDNRA,FGL2,FUT8,GFRA1,HBEGF,IGHM,IGKC,IL2RG,INHBA,ITGB3,LRRK2,MMP2,NEK7,PDGFRB,PRDM1,PTPRJ,RGS1,RGS2,SYK,TIMELESS,TIMP2,TLR4,TPSAB1/TPSB2,USP12,ZC3H12C	33	35	Cell Morphology, Humoral Immune Response, Cellular Movement
A2M,ALDOB,ATP1A2,ATP1B2,CNN1,DEF6,DES,EIF2S1,FOS,FOSB,FXYD1,GNAO1,HSPA1A/HSPA1B,IDH1,KLK8,LCAT,LRP1,MEOX2,MFGE8,MT2A,MYH11,MYOCD,NR4A1,PLCB1,PLCB4,PPP1R15A,PPP1R3C,PRKAR2B,PRKG2,PRNP,ROCK2,SCN7A,SRF,SWAP70,TRPV4	33	35	Organ Morphology, Skeletal and Muscular System Development and Function, Neurological Disease
ANTXR1,AP1B1,ATP11B,C5AR1,CAMK4,CCND2,CCR4,CD28,CEBPB,ERN1,FOXP3,GLI1,ICOS,ICOSLG/LOC102723996,IGFBP7,IL7R,KIF13A,mir-21,mir-155,MMP2,NR2F2,NRN1,ODC1,PDE3B,PDZRN3,PPAP2C,PSIP1,PTGER4,RECK,SAT1,SERPINE1,SLC7A1,STOM,TIMP3,ZYX	13	23	Cellular Development, Cellular Growth and Proliferation, Hematological System Development and Function
ASPM,B4GALNT2,CCNB2,CENPF,CEP55,DAG1,ECM1,GMNN,KIF20A,L-glutamic acid,MAPK1,MELK,MMP9,NUSAP1,PBK,PLK1,PRC1,PTGER2,STIL,TIMP1,TPX2,TTK	12	17	Cell Cycle, Cellular Assembly and Organization, DNA Replication, Recombination, and Repair
ADAM28,F13A1,FEZ1,GPNMB,IL2,IL4,IL13,KCNJ15,MN1,MS4A4A,MSMO1,MT1X,NID1,PLXNC1,RFTN1,SPINT2,TFEC,TIMP4,TNS1,TRPS1	11	16	Cell-To-Cell Signaling and Interaction, Cellular Growth and Proliferation, Hematological System Development and Function
C1GALT1,CD22,CD3D,Cdc42,CPT2,CRIM1,CRKL,CXCL10,CYR61,F11R,GLG1,HCK,IGHM,ITGB2,ITK,LTF,MS4A1,PIK3C2G,PIP5K1B,PLCG1,POU5F1,PRKD1,SELE,SELPLG,SETDB1,SH2D1B,SLAMF6,SLAMF7,SLC35C1,STYK1,SULF2,SYK,THEMIS,VAV1,VDR	11	21	Cellular Function and Maintenance, Hematological System Development and Function, Tissue Morphology
C15orf48,C5AR1,CAMP,CD34,CD80,CSF3R,DAAM1,EFNB1,EGR3,ELANE,EPHA3,ERG,GFRA3,GINS2,GPATCH2L,HPGDS,IGF1,IL17F,IL1A,IL1RN,KITLG,mir-223,MPO,MREG,MUC13,MYO1D,NFIA,PDE3A,PRTN3,PTPN11,RCAN2,SLC16A7,SLIT2,TAC1,TLR7	11	21	Cell-To-Cell Signaling and Interaction, Hematological System Development and Function, Immune Cell Trafficking
ALOX12,BPTF,CACNA1H,CCND2,Cdkn1c,CYP2J2,EGR1,GNA15,HLTF,IKZF1,IKZF3,IL18R1,MAL,MBD2,MECP2,MYO6,NAP1L2,NGFR,P2RY12,PCDH10,POLE2,PPP1R9A,PRDM1,PRNP,PROM1,RBBP4,RBBP7,REST,SELM,SGK1,SMARCA1,SMARCA4,SPIB,STAT6,XBP1	10	20	Cell Cycle, DNA Replication, Recombination, and Repair, Cellular Assembly and Organization
ACSL5,ALDOA,AQP1,ARNTL,ATF3,BHLHE22,CDH11,CDO1,COL1A2,COL4A1,CXCL12,DIXDC1,ENO3,Esrra,FASN,FBP2,GAPDH,HTT,JUP,KAL1,KCNJ8,LAMA4,LIPE,MAP2,MCAM,NR4A1,PAQR3,PEG10,PHKG1,PRDM8,PYGM,RGL1,SKIL,THBS2,ZEB1	10	20	Organismal Functions, Cardiovascular System Development and Function, Organismal Development
ACTN2,APP,ARL6IP5,ATP12A,ATP4A,C1R,CD28,ERMP1,F2RL3,FZD3,H19,HNRNPA1,HTT,KCND2,KCND3,MAGI1,MAPT,PRKAR2A,PRLR,PSEN1,RUNX1T1,TOP2A,TPD52L1	10	16	Nervous System Development and Function, Tissue Morphology, Cell-To-Cell Signaling and Interaction
ABCA6,ABLIM3,APP,ARAP2,BRCA2,BRIP1,CEBPB,DDR2,DEFB1,HERC6,IFNB1,IFNG,IL10,LPAR4,NFE2L3,SLC7A11,SOD3,STAT1,TGFB1,TGM1,TLR3,TMOD1,USP6NL,XIST	9	16	Cell Death and Survival, Cellular Development, Hematopoiesis
C1S,CCL2,CCL20,CCR6,CD207,CDH1,CTGF,CYBB,D-glucose,EDNRB,F2R,FGF12,FN1,GCNT1,ITGA1,ITGA6,ITGA7,ITGA9,ITGAE,ITGB1,ITGBL1,JUN,KITLG,MMP12,PKIG,prostaglandin E2,RGS10,RORA,RORC,RPTOR,TGFB1,TGM2,TNFRSF11A,TNFSF11,VEGFA	9	19	Cellular Movement, Hematological System Development and Function, Immune Cell Trafficking
ANXA2,CD300LD,EDN1,F2RL1,FPR2,GJC1,GLS,GSTA1,HSP90B1,HYOU1,IFIT1B,IRAK3,KLRB1,Map3k7,MMP1,MMP16,MST1R,nitrite,NR0B2,NUAK2,PI3,PPIF,PRDM1,PRTN3,RAB7B,S100A10,SLC29A1,SMAD4,TLR9,TNF,TNFAIP6,TNFRSF14,TNFSF14,TXK,XBP1	9	19	Inflammatory Response, Cell Death and Survival, Cell-To-Cell Signaling and Interaction
A2M,ADIPOR2,AIM1,ALDH1A1,CXCL1,DLL4,DUSP4,FN1,FPR2,FREM1,GDF15,GRIN1,IGHM,IL1B,IL1RN,Ins1,ITGA8,ITGA11,ITGB1,ITPKB,L1CAM,LGALS9B,LZTS2,MAPK1,MYD88,PGF,PLA2G5,PLA2G4A,PPP1R12A,RAP2A,RPS6KA3,SELE,SRR,THBS1,ZC3H12A	9	19	Organismal Injury and Abnormalities, Cellular Movement, Cell-To-Cell Signaling and Interaction
ADAM23,ADORA2A,APP,ARHGAP32,ATL2,ATP8A1,BDNF,CA12,COL4A2,ETNPPL,GRIA1,GRIN2A,GRIN2B,HTT,MEIS2,MGP,mir-132,RASD2,SGTB,TESC,TFAP2C,TPH1,WISP1	9	15	Neurological Disease, Psychological Disorders, Skeletal and Muscular Disorders
ATP,CCL3,CCL5,CENPU,CSF1,CXCL1,FABP3,GEM,GREM1,HDAC4,HTR2B,IFIT2,IFNA1/IFNA13,IL17A,IRF3,IRF7,LTBP1,MT1E,MT1F,MT1M,NEDD4L,PEA15,PIM2,PLAC8,PLD1,PRKCA,prostaglandin E2,SERPINB2,SOCS1,STARD4,STAT4,TBX21,TLR2,TLR4,TREM1	8	18	Cell-To-Cell Signaling and Interaction, Hematological System Development and Function, Immune Cell Trafficking
ADCYAP1,APP,ATP,B3GNT5,C1QL1,CKB,CRH,CTH,cyclic GMP,DCN,DHCR24,DUSP1,FOS,ganglioside GM1,GAP43,GLI3,GNRH1,HMOX2,MMP9,nitric oxide,NOS1,NPC1,NPR1,NPR2,OGN,PLAT,PLG,PTGS2,SDC1,SERPINE2,TGFB2,TLR2,UGCG,VCAN,ZFHX3	8	18	Nucleic Acid Metabolism, Small Molecule Biochemistry, Molecular Transport
BCL6,CCND2,CDH8,CRABP1,EBF1,EBI3,EOMES,EZH2,FAT3,IL2RB,IL2RG,IL4R,IL6R,IL7R,JAK3,MAP2K4,MAP3K14,MAPK8,MICAL2,mir-361,PBX3,POU2AF1,PRDM1,PTPN14,RAB27B,SMARCC1,SOCS5,ST8SIA6,STAT5A,SYTL2,TCF3,TNFRSF18,TRAF3,TRAF4,TRAT1	8	18	Cellular Development, Hematological System Development and Function, Hematopoiesis
ASL,BAG2,CD28,CNTLN,FBXL7,FOXQ1,GALNT12,GBP2,GPX7,IFNB1,IFNG,IL4,IRF1,KMT2D,MIR17HG,MYC,PCDH7,PHLPP2,RAB38,STAT1,STAT6,STXBP2,TOX3,WDR17	7	14	Cell-mediated Immune Response, Cellular Development, Cellular Function and Maintenance
ascorbic acid,CHN1,DLL1,DPYSL2,EPHA4,FGF2,FGFR1,FGFR2,FGFR3,GH1,GHRH,GHRHR,GHSR,GRIN2C,HES1,HTR1A,IGF1,IGFBP2,IGFBP3,IGFBP4,IGFBP6,KATNA1,MAP1B,Nefm,NEUROG2,NF1,NRP1,OLIG2,SDC2,SEMA3A,SNX27,SST,SSTR1,TNIK,VIM	7	17	Cellular Development, Nervous System Development and Function, Tissue Development
ACAA2,ACP5,BTK,CASP8,CCND3,CD24,CD38,CD80,CFLAR,EGR3,ELL2,F8,HSPA6,ICOS,IGJ,IL5,IL18,IL27,IL2RA,KIAA1147,LIMK2,LMO7,MAF,MAPK8,MAPK9,MUC1,NFATC1,RBPMS,SCEL,SPI1,THY1,TMEM97,TRB,TSLP,ZNF25	7	17	Hematological System Development and Function, Tissue Morphology, Cell Death and Survival
ADCY5,ADCY9,APOA1,ARPP19,BMP3,CAMK4,CASP9,CDK5,CDK5R1,CHKA,CREBBP,CYP51A1,ELK1,FMR1,GNB5,GRIN2B,H2AFX,HAP1,HIP1,HTT,ITPR1,NOS1,OPRK1,OPRM1,PCP4,PGR,PPARGC1A,PPP1R1A,PPP1R1B,PRKAA2,RAP1GAP,RGS9,SPTBN2,THRA,VCAM1	7	17	Neurological Disease, Hereditary Disorder, Psychological Disorders
AGT,AKAP6,ANTXR2,ATP2B4,Ca2+,CACNA1C,CALCB,CAMLG,CAPN3,CCK,CCKAR,CCKBR,CCL24,CCND2,CEBPD,CNR1,CXCL12,CXCL13,cyclic AMP,cyclic GMP,EFNA5,FKBP1A,FPR1,GABA,GHRH,GPR126,HTR1A,IGHM,JPH2,NOS1,RYR1,S100A4,TRPC3,TRPC6,VAV3	7	17	Cell Signaling, Molecular Transport, Vitamin and Mineral Metabolism
ABI2,ADAM19,AICDA,ASB2,BATF,CCL20,CD180,COL8A1,EGR3,ESR1,ESR2,ICOS,IKZF2,IL6,IL23R,IRF4,JAK3,KCNE2,levothyroxine,MKI67,NCOA1,NCOA2,NOTCH1,PRDM1,RORA,RORC,SEMA3C,SULF1,SULT1C2,TC2N,THRB,TIGIT,TNFSF13,TSHB,XBP1	7	17	Inflammatory Disease, Inflammatory Response, Neurological Disease
APP,BTNL2,CD86,CD109,CDKN2B,CDKN2C,CEBPB,CSF2,CYP2S1,DAPL1,DAPP1,FOXO1,GJB2,GPD2,GPR34,IFITM3,IFNG,IKZF2,IL2,IL17A,IL1B,IRF5,MAVS,MYO10,NDN,RRAGD,SELL,SMPD2,ST14,TCF3,TGFB1,TNF,XDH	7	16	Inflammatory Response, Cell Death and Survival, Cell-To-Cell Signaling and Interaction
AGL,ARHGAP21,CREM,CRYAB,CYP27A1,DNASE2,DOCK8,GAS1,GBP6,GLP2R,HMGCR,HMGCS1,IFIT3,IFNAR1,IFNB1,IRF3,IRF5,IRF7,MAPK13,MAVS,MPZ,NFATC2,NFIX,NPTX1,OAS1,PKP2,SAMSN1,SASH1,SERT2,SOCS1,SQLE,STAT2,THBS2,TLR3,VCL	6	16	Infectious Disease, Antimicrobial Response, Inflammatory Response
AQP3,CAMK4,CCL2,CD14,CD55,CYP2E1,DIO2,DMBT1,DRD2,DYSF,EGR1,EGR2,ethanol,FOS,GABA,GAD2,GRIN2A,GZMB,Gzmc,HMCN1,ID4,IFIT1,IL15,IL1R2,JUN,MAP2,MT1G,NCAM1,NLGN2,PLCL1,PRF1,SEC23A,SRGN,TFRC,YARS	6	16	Neurological Disease, Skeletal and Muscular Disorders, Hereditary Disorder
BTBD11,CAMK2A,CAMK2B,CASK,CCNE2,CDCA8,corticosterone,DLG3,DLG4,DLGAP2,DSTN,FMR1,FYN,GRIK2,GRIK5,GUCY1A2,GUCY1B3,IQSEC1,KCNJ10,KCNJ16,MECP2,MSRB2,NGF,NLGN1,NRXN1,NTRK2,PSEN1,SLC25A4,SLC4A4,SNRPN,SOX9,SRC,SYT1,TUBB6,UBE3A	6	16	Cell-To-Cell Signaling and Interaction, Nervous System Development and Function, Behavior
ACKR3,AQP1,AR,ATP1B1,CASQ2,CD28,CD38,CD40LG,CDH5,Cdkn1c,CELF2,CLCN3,CSF2,CSGALNACT2,HN1,HTT,IFNG,IGFBP5,IL2,IL4,IL5,IL15,JUNB,MMD,NR3C1,ODC1,PRKAG3,RNF128,SOD1,TGFB1,TREM1,TSPAN8,UGT1A9 (includes others),VAMP8,WNK2	6	16	Cell-To-Cell Signaling and Interaction, Hematological System Development and Function, Immune Cell Trafficking
CA9,CCNG2,CD40LG,CD79A,CD79B,CDC6,CDK2,CYTH1,CYTIP,E2F2,E2F3,E2F4,EBI3,GOT1,GPR183,HIF1A,HK2,IGHA1,IGHG1,MYC,POU2AF1,PTPRG,RASSF2,RBL1,RBL2,RGS1,RRM2,RUVBL1,RUVBL2,SEMA4D,SLC38A1,TNFAIP2,TNFRSF13C,TNFSF13,UBD	6	16	Cell Morphology, Hematological System Development and Function, Tissue Morphology
AKT3,CD247,CLEC4G,CYBA,DICER1,ELF4,ERBB2,FPR2,GNB4,IFNG,IL4I1,ITCH,KLF4,KLK7,KLRB1,LPAR3,mir-21,mir-143,NCR1,norepinephrine,NR2F6,PDE5A,PTPRN2,RNF128,RUNX3,SEMA7A,SLC16A2,SLC7A5,SLC7A8,SST,TARDBP,TNFRSF18,TP63,TRD,TRG	6	16	Cellular Development, Cellular Growth and Proliferation, Hematological System Development and Function
Ccl2,CD40,CD69,CD86,CEACAM1,CEACAM5,CSF2,CTNNB1,GADD45B,GPT2,ICAM1,IL5,IL6,IL18,IL17A,IL2RA,LPIN2,LRG1,LRRN3,MYD88,MYRF,NTN1,PAX6,PDCD1,PTGS2,SAMHD1,SATB1,SELL,SLC27A2,STAT1,STAT6,TBC1D9,TICAM1,TMEM2,UNC5B	6	16	Cellular Movement, Hematological System Development and Function, Immune Cell Trafficking
ACTN1,AKT1,APC,Ccl2,CD44,CD3E,CDK5,CORO1C,CTNNB1,DIAPH1,EZR,FZD7,GPR37,IL23A,IVNS1ABP,KIF11,LCN2,MRC1,MSN,NF2,NOS2,PARK2,PTEN,PTPN22,RAC1,RDX,RELA,SATB1,SLC6A3,SLC9A3R1,SNCA,SPN,TARDBP,TBC1D1,VCP	6	16	Cell Death and Survival, Cellular Movement, Cancer
AGT,AGTR1,ALDH1A2,beta-estradiol,BIRC5,BMP2,BTC,C4A/C4B,CPT1B,DKK1,DRD1,DRD2,EFEMP1,ESCO2,FRMD6,GAL,GHRH,HOXA4,HOXD4,HSD11B1,IGF1,IGF2,IGFBP2,NBN,OCLN,PDK4,POU1F1,PRL,PTGFRN,PTGIS,PTTG1,RARB,RASSF8,TP53,WNT7A	6	16	Tissue Morphology, Embryonic Development, Organismal Development
APBB1,APP,AVPR1A,Ca2+,CAP2,CD27,CHRDL1,CNKSR2,DGKB,DLG4,ETS1,GABRA5,GRM5,HDAC1,HDAC2,HDAC3,HDAC4,HDAC9,HOMER1,HOMER2,HTT,IL4,IL13,K+,L-glutamic acid,MYOG,NBEA,PPP3CA,PRKCB,PTGFR,TNF,TRPC1,TSHZ3,TSPAN13	6	15	Amino Acid Metabolism, Cell-To-Cell Signaling and Interaction, Molecular Transport
ANXA6,CA2,CD38,CD40,CD83,CD40LG,CEBPB,COL6A3,CSF1,CYSLTR1,CYSLTR2,F2RL2,FGF7,FOXP3,GLIPR2,GPM6B,IGHG3,IL4,IL5,IL13,IL19,IL17A,IL1RL2,NFATC1,PKP3,PNP,PRKG1,PROS1,PSEN1,PTBP2,S1PR1,STAT6,TFEC,TNFSF8,TP53	5	15	Cell-To-Cell Signaling and Interaction, Hematological System Development and Function, Cellular Growth and Proliferation
ADAMTS1,ATP1A1,BMPR1B,BOC,BUB1,Cacnb1,CACNB2,CALCB,CARTPT,CD209,CDK5,CLU,dopamine,DSG3,FGF8,FGF19,FTH1,GDNF,glutathione,GUCY1A3,HSF1,HSF2,JUN,KCNK1,KCNN3,MAOA,MAOB,mir506,MYOM1,NMU,NPY,NR4A2,SHH,SOD1,XIAP	5	15	Cell-To-Cell Signaling and Interaction, Drug Metabolism, Molecular Transport
ACADM,ARG1,BCL11B,BMP1,CFD,EIF4EBP1,EMP3,ESRRG,Foxp1,GFAP,HIP1,HTT,IER3,ILK,inosine,KCNIP2,KLF5,MAP3K8,mir-29,MPZ,MYL9,NGEF,PMP22,Pou3f1,PPARGC1B,PPP1R1B,PPP3CA,SAMD4A,SATB2,SERPING1,SKI,TSPO,UCP2,WFS1,ZC3H6	5	15	Hereditary Disorder, Neurological Disease, Psychological Disorders
ACHE,ADAM10,APLP1,BACE1,BDH1,CDH2,CHAT,CNTF,CNTFR,DCLK1,DPYSL2,FOXA1,GJA1,GJB6,HTR2C,INADL,LRP1,MAP6,MBP,MPDZ,MYH10,NOS1,NR4A2,PER3,PSEN1,QKI,RGS4,STX2,STXBP1,SYP,TGFBR2,TH,Tpm1,TPM3,YWHAZ	5	15	Cellular Development, Nervous System Development and Function, Tissue Development
ANK3,CDK5,CNTN1,CNTNAP1,CTNNB1,CTNNBIP1,CTSF,DKK1,FGF5,FRZB,GAP43,HDAC4,HDAC9,HEPH,HOOK1,JUN,MBP,MPZ,NFASC,NGFR,NRCAM,NTF3,NTRK1,PTN,PTPRZ1,quinolinic acid,RTN4,SOX2,SPTBN4,TNC,TSPAN2,UGT8,ULK2,VEGFA,WNT3A	5	15	Cellular Development, Nervous System Development and Function, Tissue Development
ADCYAP1,ADCYAP1R1,AGER,AKAP12,ANGPT1,BMPR1A,CD80,CD247,CNTNAP2,CTSE,CXCL3,EBI3,EPB41,GAP43,GFAP,GYS2,IL10,IL25,IL17RB,IL23A,IRF1,ITGAM,JAK1,LAMP2,MAN1A1,MOG,NEDD4,nitric oxide,NOTCH1,P2RY2,SCARB1,SERPINB1,SMPDL3B,SNCA,superoxide	5	14	Cellular Movement, Immune Cell Trafficking, Hematological System Development and Function
ACTG1,AQP4,BMPR2,CAV3,DAG1,Dmd,DMD,DTNA,EGR2,ERBB2,ERBB3,FBXO32,FKRP,FOXO3,KCNJ10,LAMA2,LAMC1,MBP,mir-130,MYOC,NOS1,PRX,SGCA,SGCB,SGCD,SGCE,SGCG,SNTA1,SNTB1,SNTB2,SSPN,TRIM63,UTRN	4	13	Developmental Disorder, Skeletal and Muscular Disorders, Hereditary Disorder
acetylcholine,ACHE,adenosine,BCHE,BDNF,CADPS2,CDON,CHAT,CHGA,CHRM2,CNR1,CNTF,DLG1,DNAJB5,DRD1,DRD2,DUSP1,GABA,GDNF,GHRL,GPRASP1,GRB14,HCN1,IGF1R,MAP3K3,MAPK14,NCS1,NGF,PLG,PPP1R1B,PRIMA1,SLC17A6,SLC6A4,TRPA1,TUBB3	3	12	Behavior, Cellular Compromise, Neurological Disease

Figure legends

Figure S1: Heatmap of differentially expressed genes between achalasic and control sample tissues. Samples are grouped by hierarchical clustering.

Figure S2: Graph connecting network-eligible molecules as calculated by IPA and over-representing the cell morphology, humoral immune response, and cellular movement processes. The achieved score of the graph was 33, while the sparseness ρ value was 5.25.

Figure S3: Graph connecting network-eligible molecules as calculated by IPA and over-representing the organ morphology, skeletal and muscular system development processes, and neurological disease processes. The achieved score of the graph was 33, while the sparseness ρ value was 6.01.

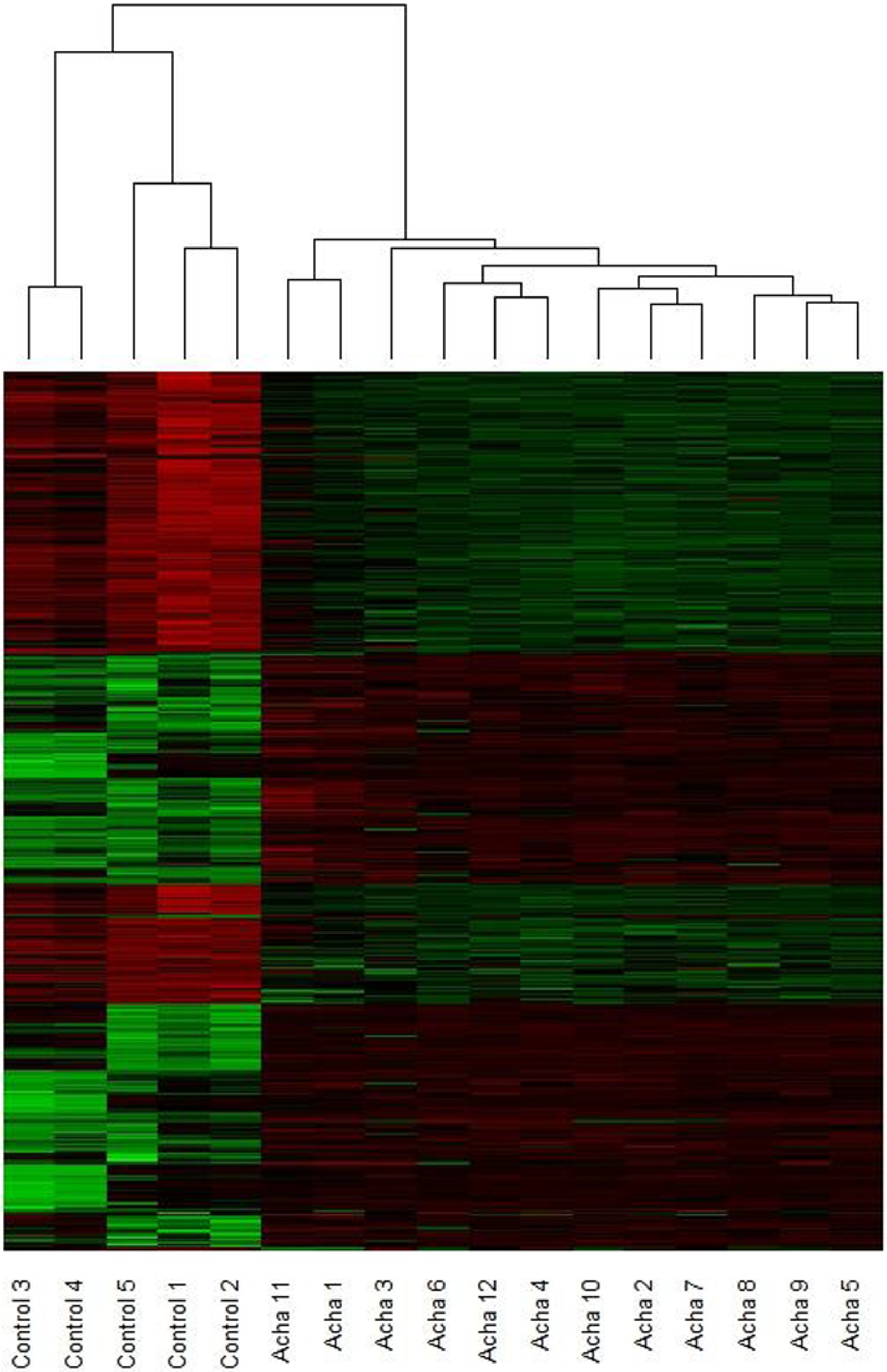
Control 3
Control 4
Control 5
Control 1
Control 2
Acha 11
Acha 1
Acha 3
Acha 6
Acha 12
Acha 4
Acha 10
Acha 2
Acha 7
Acha 8
Acha 9
Acha 5

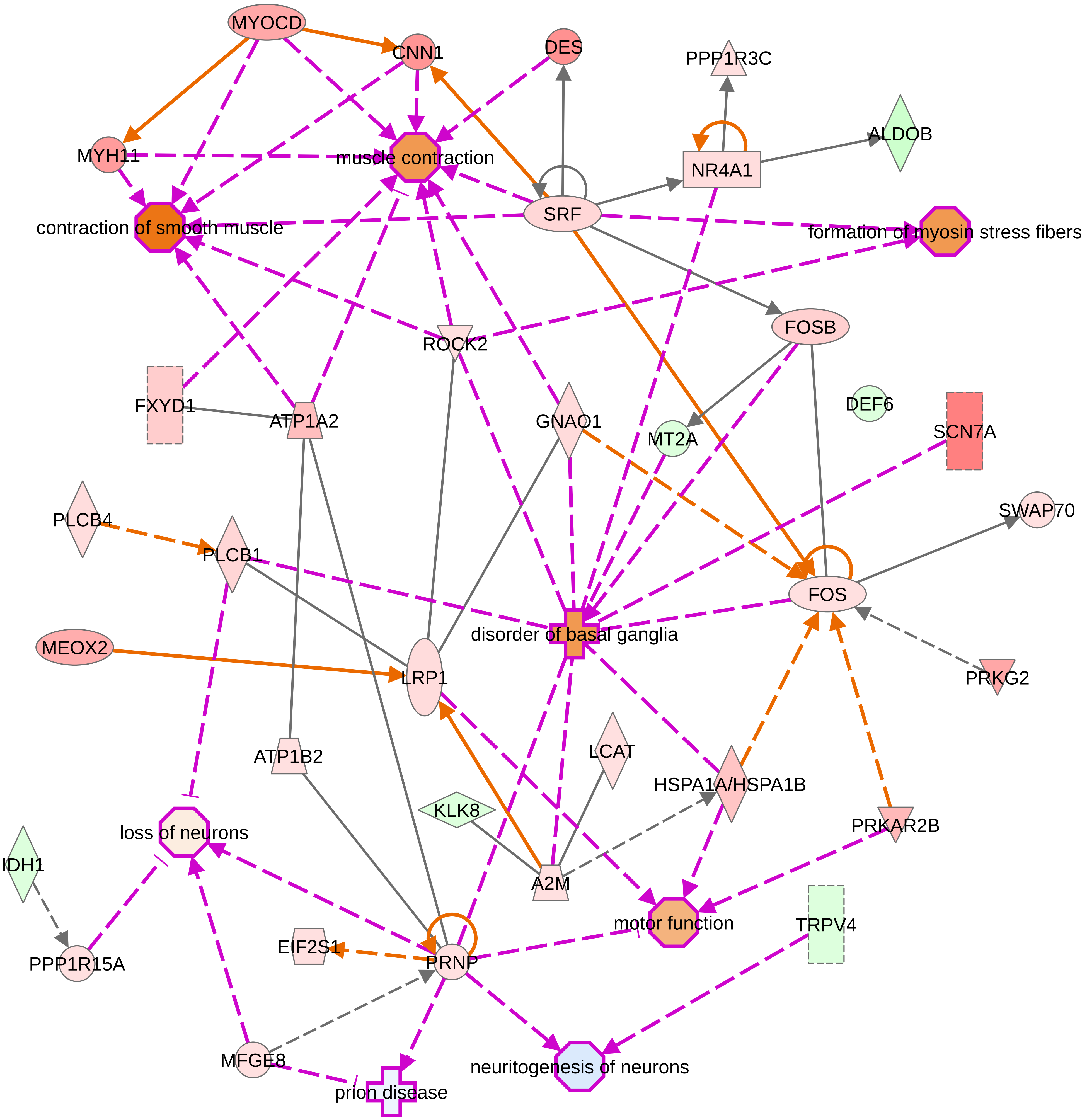

MYOCD
CNN1
DES
PPP1R3C
MYH11
muscle contraction
NR4A1
ALDOB
SRF
contraction of smooth muscle
formation of myosin stress fibers
FOSB
ROCK2
FXYD1
ATP1A2
GNAO1
MT2A
DEF6
SCN7A
SWAP70
PLCB4
PLCB1
FOS
disorder of basal ganglia
MEOX2
LRP1
PRKG2
ATP1B2
LCAT
HSPA1A/HSPA1B
KLK8
PRKAR2B
loss of neurons
IDH1
A2M
motor function
TRPV4
EIF2S1
PRNP
PPP1R15A
MFGE8
neuritogenesis of neurons
prion disease

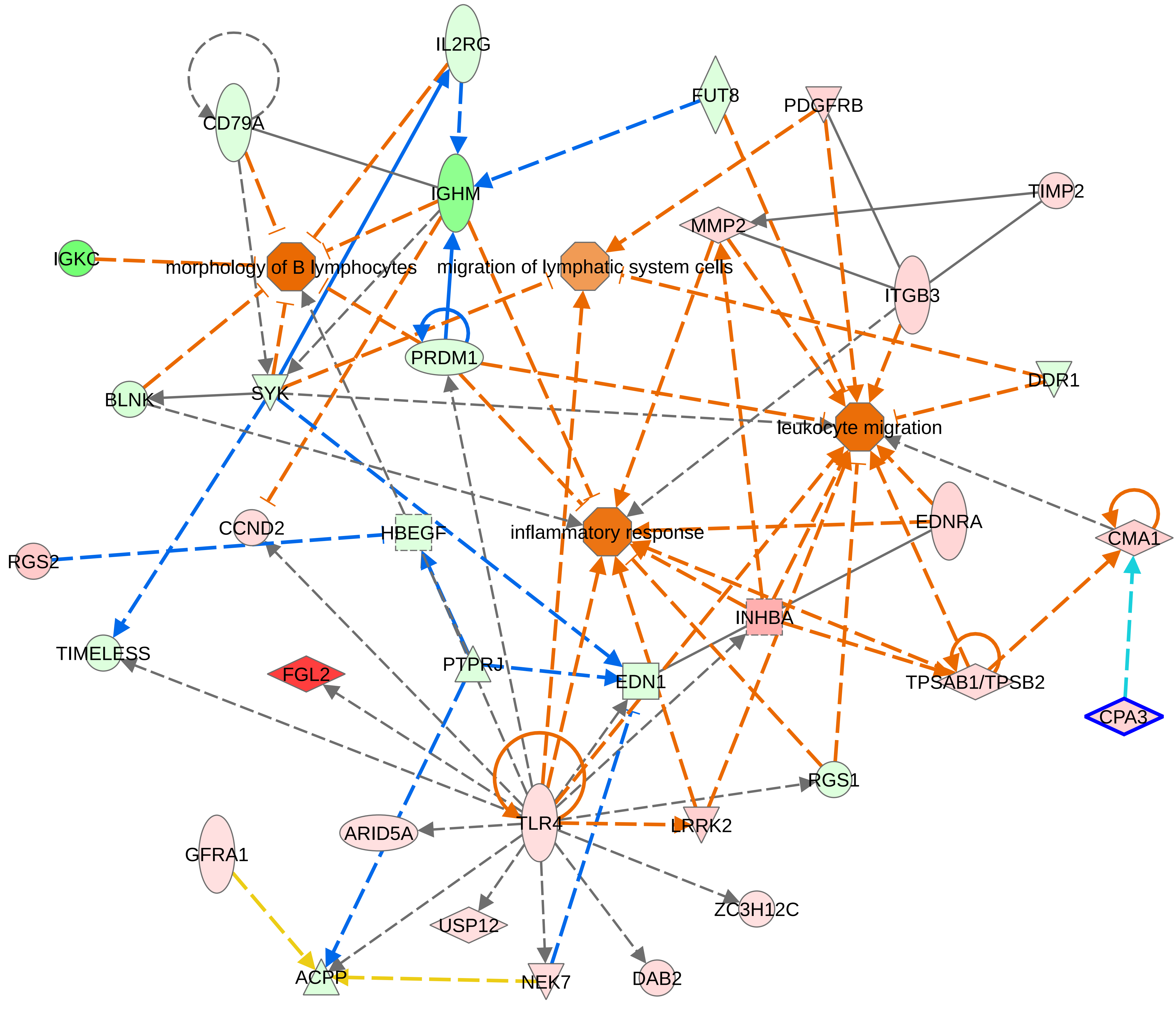
IL2RG
CD79A
FUT8
PDGFRB
IGHM
TIMP2
MMP2
IGKC
morphology of B lymphocytes
migration of lymphatic system cells
ITGB3
PRDM1
BLNK
SYK
DDR1
leukocyte migration
CCND2
HBEGF
inflammatory response
EDNRA
CMA1
RGS2
INHBA
TIMELESS
FGL2
PTPRJ
EDN1
TPSAB1/TPSB2
CPA3
RGS1
ARID5A
TLR4
LRRK2
GFRA1
ZC3H12C
USP12
ACPP
NEK7
DAB2
